# Pattern Recognition Proteins: First Line of Defense Against Coronaviruses

**DOI:** 10.3389/fimmu.2021.652252

**Published:** 2021-09-23

**Authors:** Carlos A. Labarrere, Ghassan S. Kassab

**Affiliations:** California Medical Innovations Institute, San Diego, CA, United States

**Keywords:** pattern recognition proteins, SARS-CoV-2, COVID- 19, acute respiratory distress syndrome, ATH - atherosclerosis, surfactant proteins A and D (SP-A and SP-D), C-reactive protein, innate and adaptive IgM antibodies

## Abstract

The rapid outbreak of COVID-19 caused by the novel coronavirus SARS-CoV-2 in Wuhan, China, has become a worldwide pandemic affecting almost 204 million people and causing more than 4.3 million deaths as of August 11 2021. This pandemic has placed a substantial burden on the global healthcare system and the global economy. Availability of novel prophylactic and therapeutic approaches are crucially needed to prevent development of severe disease leading to major complications both acutely and chronically. The success in fighting this virus results from three main achievements: (a) Direct killing of the SARS-CoV-2 virus; (b) Development of a specific vaccine, and (c) Enhancement of the host’s immune system. A fundamental necessity to win the battle against the virus involves a better understanding of the host’s innate and adaptive immune response to the virus. Although the role of the adaptive immune response is directly involved in the generation of a vaccine, the role of innate immunity on RNA viruses in general, and coronaviruses in particular, is mostly unknown. In this review, we will consider the structure of RNA viruses, mainly coronaviruses, and their capacity to affect the lungs and the cardiovascular system. We will also consider the effects of the *pattern recognition protein (PRP) trident* composed by (a) Surfactant proteins A and D, mannose-binding lectin (MBL) and complement component 1q (C1q), (b) C-reactive protein, and (c) Innate and adaptive IgM antibodies, upon clearance of viral particles and apoptotic cells in lungs and atherosclerotic lesions. We emphasize on the role of pattern recognition protein immune therapies as a combination treatment to prevent development of severe respiratory syndrome and to reduce pulmonary and cardiovascular complications in patients with SARS-CoV-2 and summarize the need of a combined therapeutic approach that takes into account all aspects of immunity against SARS-CoV-2 virus and COVID-19 disease to allow mankind to beat this pandemic killer.

## Introduction

**Figure 1:**
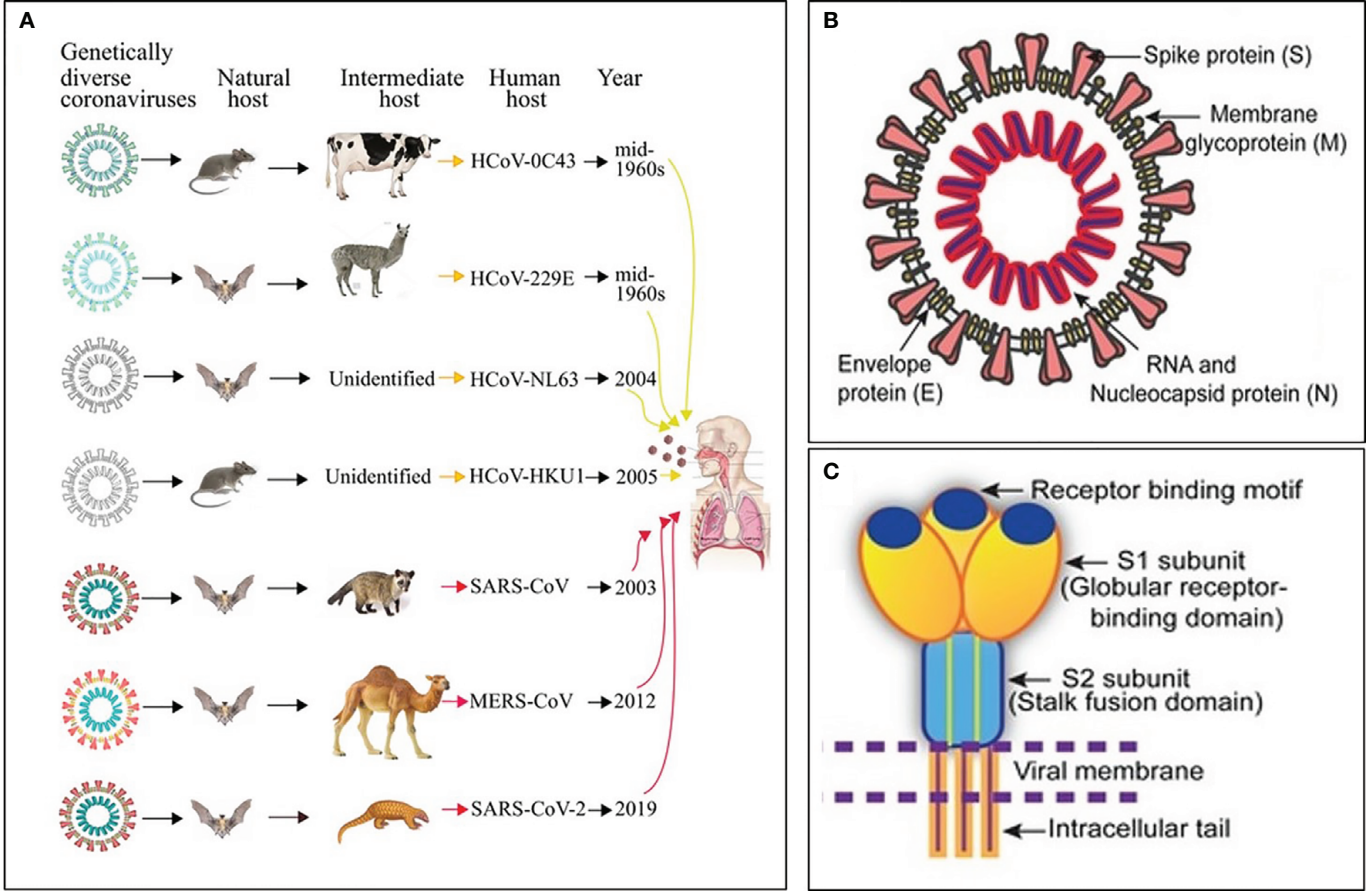
Origin, transmission and structure of pathogenic human coronaviruses (HCoVs). **(A)** Yellow and red arrows represent mild and severe infections in humans, respectively. **(B)** Schematic diagram of the SARS-CoV structure. **(C)** Cartoon showing key features and the trimeric structure of the SARS-CoV-2 S protein. Modified from Mittal et al., (5).

Ribonucleic acid (RNA) viruses (orthomyxoviruses and coronaviruses) have been the origin of many epidemics and pandemics over the years, such as H1N1, severe acute respiratory syndrome coronavirus (SARS-CoV) and Middle East respiratory syndrome coronavirus (MERS CoV). One hundred years after the 1918-1919 catastrophic and historic influenza A pandemic (1–3), the world is facing another pandemic due to the SARS-CoV-2 (4) (**Figure 1A**). Currently, there is no specific drug or vaccine against this deadly virus; therefore, there is a pressing need to understand the mechanism(s) through which this virus enters the host cell (5). Coronaviruses (CoVs), named for the crown-like spikes on its surface, are enveloped viruses containing single-stranded, non-segmented, positive sense RNA genetic material (5–12). On their surface, coronaviruses have club-like protrusions constituted by the trimeric spike (S) glycoprotein. The coronavirus RNA genome encodes spike (S), envelope (E), membrane (M), and nucleocapsid (N) structural proteins, common to SARS-CoV, MERS-CoV and coronavirus disease 2019 (COVID-19) (**Figure 1B**) (13). The SARS-CoV and MERS-CoV genomic RNA is inside the N protein, while the M, E, and S proteins conform the envelope that surrounds the capsid (**Figure 1B**). The human CoVs S protein (**Figure 1C**) facilitates host cell virus entry and subsequent membrane fusion allowing viral infection (13). The Class I viral S protein can be cleaved into two functional subunits: 1) An amino-terminal S1 subunit, and 2) A carboxyl-terminal S2 subunit. The S1 subunit causes virus–host cell receptor binding to the cell surface receptor angiotensin-converting enzyme 2 (ACE2) receptor through the receptor binding domain and the S2 subunit allows virus–host membrane fusion (**Figure 1C**). SARS-CoV-2 is then taken up into the target cell through endocytosis. The viral envelope contains the (a) M glycoprotein (most abundant structural protein embedded in the envelope through three transmembrane domains), and (b) Small E transmembrane protein being in the envelope in low amounts (14). The helically symmetric nucleocapsid is formed by the binding of N protein to the RNA genome in a beads-on-a-string fashion.

Seven CoVs are known to infect humans and cause respiratory diseases with mild to severe outcomes, while other CoVs infect animals such as pigs and chickens (14) (**Figure 1A**). CoVs are recognized as one of the viral causes of the common cold. Among all CoVs presently identified, SARS-CoV in 2002 to 2003, and MERS-CoV in 2012, are zoonotic and highly pathogenic provoking regional and global outbreaks (14). The high mortality rate in both pandemics (~9.6% in SARS-CoV and ~35% in MERS-CoV) was associated with the development of fatal respiratory failure and acute respiratory distress syndrome called ARDS (13, 14). Since at present, no vaccine or specific antiviral drug are available for either SARS-CoV or MERS-CoV, it is paramount to 1) Enhance the host immune responses, 2) Improve the body’s capacity to neutralize virus particles, and 3) Intensify removal of virus particles by the host macrophages to avoid virus-mediated cell invasion, damage and death; i.e., modulate the inflammatory response (15).

COVID-19 originated by the SARS-CoV-2 has reached a pandemic level (16). It was first identified in Wuhan, China in December 2019 and has infected almost 204 million and caused more than 4.3 million deaths worldwide with a 2.1% fatality rate (17). Two systems in the body are directly implicated in disease severity and mortality: respiratory and cardiovascular; since compromise of lungs, heart and vasculature significantly worsen outcome (18). The SARS-CoV-2 family of viruses mutates easily and infects mostly bats, pigs, small mammals, and humans. Recently, these viruses have become growing players in infectious-disease outbreaks around the world. COVID-19 affects the lower respiratory tract of the lung airways, mainly the alveoli where the exchange of oxygen and carbon dioxide occurs during respiration. This leads to respiratory distress due to alveolar damage accompanied by immunopathological lesions as the most frequent cause of death. Patients initially develop flu-like symptoms that can advance to development of shortness of breath and pneumonia-related complications necessitating a respirator. People of all ages have been infected, but individuals at greatest risk of serious illness are older individuals with chronic lung disease, diabetes, cancer, and/or cardiovascular disease (18–25). Clinical outcomes are worse in patients with COVID-19 having cardiovascular disease and risk factors such as hypertension, diabetes, and obesity. For example, patients with a long history of coronary artery disease and patients with risk factors for developing atherosclerotic cardiovascular disease have a markedly greater risk of developing an acute coronary syndrome during acute infections, which has also been found in epidemiologic and clinical studies of influenza (26–28) and other acute inflammatory disorders (19, 29). Chronic inflammation of the vessels associates with arterial intimal cholesterol-laden macrophages (foam cells), which increase the possibility of acquiring a severe COVID-19 infection (30, 31). A chronic inflammatory disease like atherosclerosis may be an ideal environment for SARS-CoV-2 to reproduce due to its high viral replication capacity in human cells which can lead to immune system dysregulation and hyperactivation of pro-inflammatory pathways with subsequent cytokine release contributing to the cytokine storm (30, 31). This process can accelerate the progression of the disease and predispose the arteries to further develop atherothrombotic complications (30, 31).

SARS-CoV-2 is particularly different from SARS-CoV and MERS-CoV regarding global epidemiology and its impact on the cardiovascular system (18). COVID-19 is associated with an elevated inflammatory load that can lead to vascular inflammation and cardiac injury (16, 19). Acute cardiac injury, evidenced by elevations in cardiac troponin levels, is found in 8-62% of patients hospitalized with COVID-19 and is associated with greater disease severity, necessity for mechanical ventilation, and death (18). As described with other coronaviruses, SARS-CoV-2 can induce an enormous release of numerous cytokines and chemokines (20, 32, 33) that can lead to vascular and myocardial inflammation and plaque instability (19). Circulating cytokines released during a severe systemic inflammatory response may lead to instability and rupture of atherosclerotic plaques. Coronary injury (e.g., coronary artery plaque inflammation, acute coronary syndrome and myocardial infarction) and failure have also been reported during the MERS-CoV pandemic (16, 33). Patients at risk of myocardial injury are older and have higher levels of hypertension, coronary artery disease, heart failure, and diabetes than patients with normal levels of Troponin I or T. Patients with COVID-19 and myocardial injury also have more severe systemic inflammation, with increased leukocyte counts and elevated levels of C-reactive protein (CRP), procalcitonin and other biomarkers of myocardial injury and stress, like creatine kinase, myoglobin, and N-terminal pro-B-type natriuretic peptide (16, 19, 33–36). Patients who develop COVID-19-associated cardiac injury have increased incidence of acute respiratory distress syndrome and need for assisted ventilation than patients without myocardial injury.

As SARS-CoV-2 enters the lung cells through the binding of viral S protein to ACE2 (the functional receptor of SARS-CoV-2), it initiates replication. Although ACE2 is ubiquitous, it is more often expressed by lung epithelial cells, cells of the vascular system, and myocytes (37–41). The body recognizes all viruses as foreign invaders triggering an immune response to halt replication. The immune response to SARS-CoV-2 can also damage lung tissues through severe inflammation complicating pneumonia. Pneumonia causes alveoli to become inflamed and filled with fluid, making it harder to breathe and deliver oxygen to blood, potentially triggering a cascade of respiratory/cardiac complications. The lack of oxygen leads to more inflammation which may lead to further complications such as severe liver and kidney damage, and even death. Patients often must be placed on ventilators for weeks as they recover from the viral infection. As the number of patients requiring respirators surpasses the number of ventilators available in some hospitals and ICUs, the need for treatment halting the progression of disease becomes urgent. Pneumonitis in severe COVID-19 disease shows significant capillary injury with mural and luminal fibrin deposits, and neutrophil infiltration accompanied by deposits of complement components C4d and C5b-9 in the microvasculature (42). Severe inflammation may lead to a catastrophic microvascular injury syndrome mediated by a procoagulant state and complement activation (42). Endothelial injury can be directly generated by viral replication into the host cells and by ACE2 downregulation, that exposes cells to angiotensin 2 in the absence of the modulator effects of angiotensin 1–7 (37–41).

**Figure 2:**
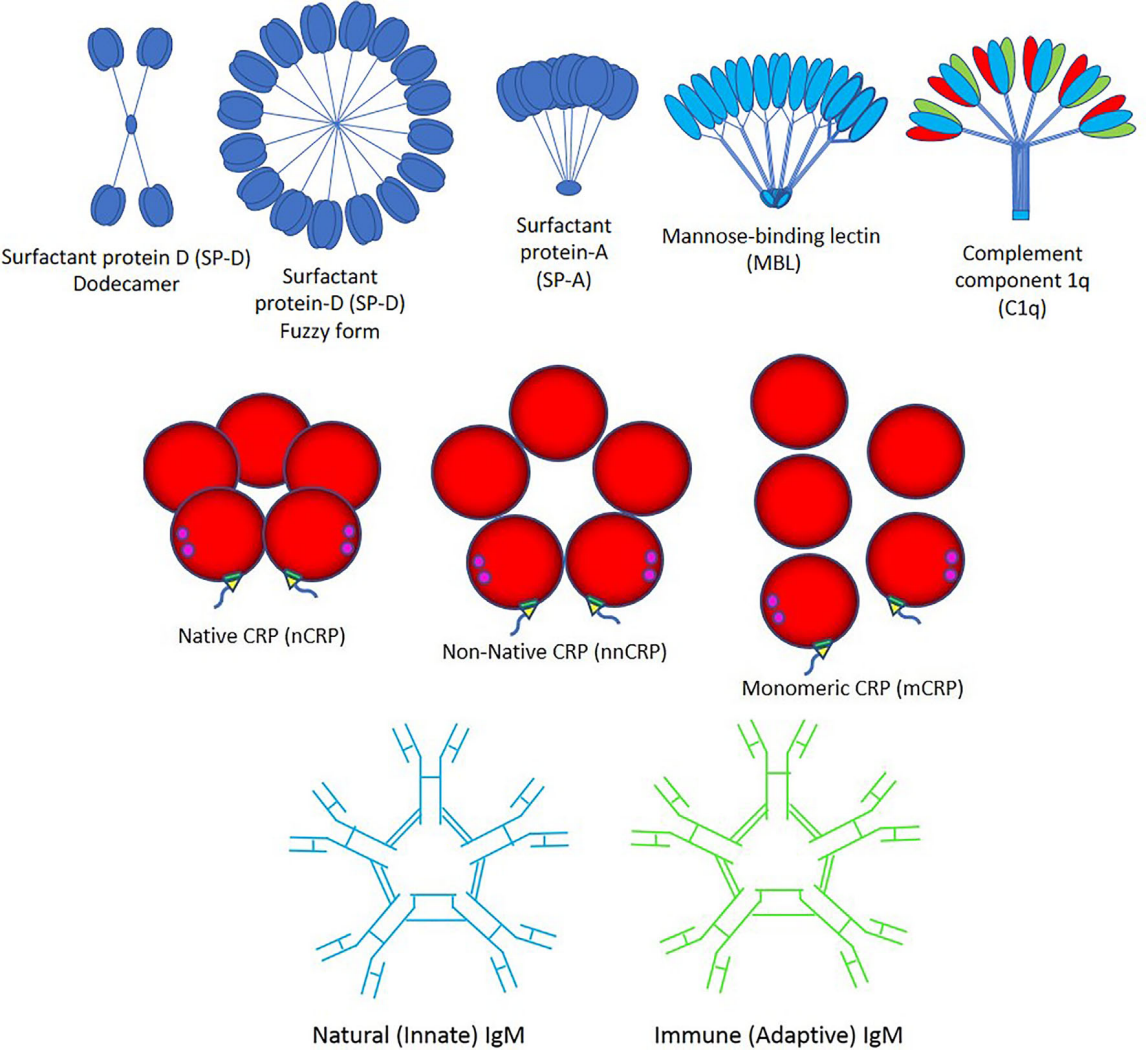
Pattern recognition proteins. The trident of pattern recognition proteins is composed by: (Top) surfactant proteins D and A (SP-D and -A), mannose-binding lectin (MBL) and complement component 1q (C1q); (Middle) native C-reactive protein (nCRP), non-native CRP (nnCRP) and monomeric CRP (mCRP); and (Bottom) natural (innate) immunoglobulin M (IgM) and immune (adaptive) IgM.

Importantly, viruses can induce an apoptotic cell death response at the end of their infectious cycle. During apoptosis, the cellular contents including the progeny virions are wrapped into plasma membrane-bound apoptotic bodies (the hallmark of apoptosis) which are quickly taken by surrounding cells. This impedes the development of an inflammatory response that allows the spreading of the infection without being detected (43). It was recently demonstrated that apoptotic bodies generated from influenza A-infected monocytes contain viral RNA, proteins, and virions promoting both viral propagation and antiviral immune response (44). Three major pathways such as apoptosis, necroptosis, and pyroptosis likely induce cell death in SARS-CoV-2-induced COVID-19 disease, directly through virus cell invasion or indirectly through intense cytokine and chemokine release causing immune cell damage (45–54). SARS-CoV-2 promotes inflammation and could potentiate or accelerate the pre-existing systemic inflammatory state of individuals with obesity, diabetes, hypertension and other inflammatory diseases, through NOD-like receptor (NLR) family pyrin domain-containing 3 (NLRP3) inflammasome activation *via* cleavage and activation of key inflammatory molecules including active caspase-1 (Casp1p20), interleukin (IL)-1β, and IL-18 and cell release of those pro-inflammatory cytokines through Gasdermin D pores commonly found in cell death by pyroptosis (45–54). Viral particles need to be cleared by macrophages with the help of the *pattern recognition protein (PRP) trident* composed by (a) Surfactant proteins A and D, mannose-binding lectin (MBL) and complement component 1q (C1q); (b) Native C-reactive protein (nCRP), non-native CRP (nnCRP) and monomeric CRP (mCRP); and (c) Natural (innate) immunoglobulin M (nIgM) and immune (adaptive) IgM (iIgM) (**Figure 2**).

## Surfactant Proteins, Mannose-Binding Lectin, Complement 1q, and the Coronavirus

**Figure 3:**
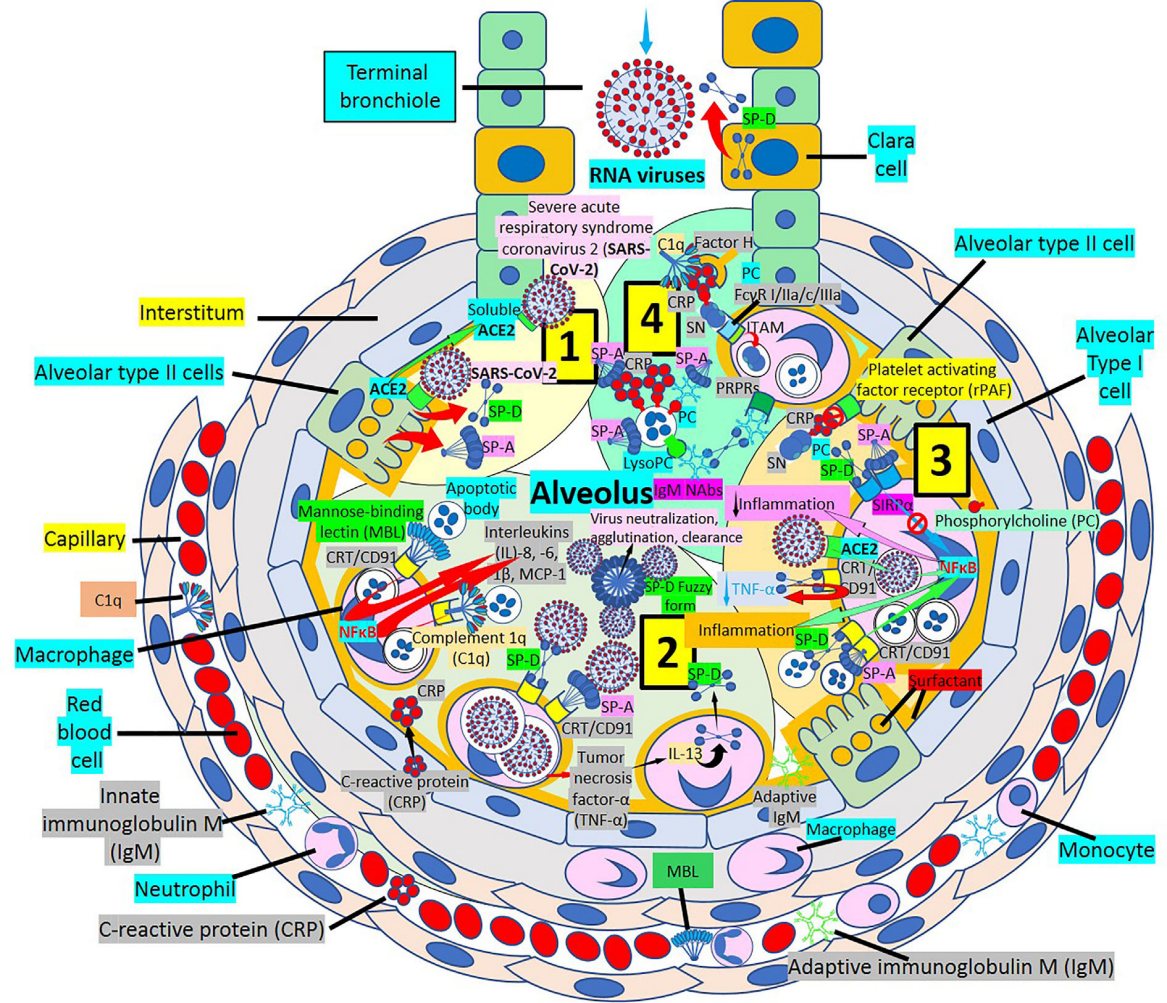
Alveolar surfactant protein innate immune response to RNA viruses. [1] Intra-alveolar RNA viruses are exposed to surfactant proteins (SP)-A and -D produced by alveolar type II (AII) and Clara cells. SARS-CoV-2 binds ACE2 receptors in AII cells releasing soluble ACE2 to protect AII cells against SARS-CoV-2 infection. [2] MBL and C1q bind calreticulin/CD91 (CRT/CD91) receptors enhancing apoptotic body macrophage uptake and nuclear factor κB (NFκB)-mediated intra-alveolar release of IL-1ß, -6, -8 and monocyte chemoattractant protein-1 (MCP-1). CRT/CD91 bound SP-A and SP-D facilitate virus phagocytosis. TNF-α release stimulates IL-13-mediated SP-D macrophage output; and SP-D fuzzy form contributes to virus neutralization, agglutination and clearance. Circulating innate and adaptive IgM, MBL, C1q and CRP; and alveolar type I cell CRP synthesis and release contribute to alveolar immune defense. [3] CRT/CD91-bound SP-A and SP-D facilitate apoptosis and activate NFκB-mediated inflammation. Macrophage ACE2 facilitates virus uptake and SP-D-CRT/CD91 binding reduces TNF-α release. Signal inhibitory regulatory protein α (SIRPα) SP-A and SP-D binding inhibits inflammation. Native CRP blocks PC-mediated attachment of *streptococcus pneumoniae* (SN) to rPAF on alveolar epithelial cells. PC is a main component of alveolar surfactant. [4] Interaction of SP-A, SP-D, CRP and IgM natural antibodies (NAbs) with apoptotic cells facilitate macrophage uptake through pattern recognition protein receptors (PRPRs). CRP bound to C1q and factor H mediates SN uptake *via* Fcγ receptors and cytoplasmic immuno-tyrosine activating motif (ITAM) activation.

Surfactant, a complex of 90% phospholipids (mostly dipalmitoylphosphatidylcholine), and 10% proteins, (mainly surfactant proteins (SP)-A, B, C and D) that lines the entire alveolar epithelium (**Figure 3**), plays a critical role in pulmonary innate immune defense (55, 56). Multimeric SP-A and SP-D are pattern recognition proteins and members of the collagen-containing C-type lectin (*collectin*) family that bind to repetitive carbohydrate moieties commonly found on the surface of viruses and other microbes in a Ca^2+^-dependent and carbohydrate-specific manner. This leads to virus agglutination and enhanced macrophages and neutrophil phagocytosis and killing (57, 58). Pulmonary surfactant is produced by alveolar type II cells expressing ACE2 receptors and are a direct target for SARS-CoV-2 viral invasion. Indeed, ACE2 expression is predominantly observed in alveolar type II cells and alveolar macrophages and is strongly upregulated with age and alveolar damage associated with mechanical ventilation, being a potential mechanism for the increased mortality in patients with severe COVID-19 (59). After entry into the lungs, SARS-CoV-2 is proposed to destroy type II alveolar cells, the site for the synthesis of pulmonary surfactants, resulting in decreased production of pulmonary surfactant and leading to development of acute respiratory distress syndrome (60). Soluble ACE2 could be protective against SARS-CoV-2 since it has been found that the proteolytic cleavage-induced shedding of soluble ACE2 could protect against SARS-CoV-2 virus infection of alveolar type II cells (61–65) (**Figure 3**).

SP-A and SP-D direct interaction with various viruses more frequently results in viral neutralization and enhanced phagocytosis (57). SP-A and SP-D inhibit the hemagglutinin binding activity of influenza A virus; and SP-D also lowers neuraminidase activity (57). SP-D is a more potent inhibitor of influenza A virus infectivity than SP-A which induces massive aggregation of influenza A virus particles, inhibits hemagglutinin and neuraminidase activities, and neutralizes viral particles (57). Multivalent lectin-mediated interactions of SP-D with influenza A viruses result in viral aggregation, reduced epithelial infection, and enhanced virus clearance by phagocytic cells (66). SP-D has strong anti-influenza A viral activity through the binding of its carbohydrate recognition domain region to carbohydrates (mannosylated, N-linked) on viral hemagglutinin and neuraminidase (57). SP-D recognizes and binds the SARS coronavirus spike glycoprotein and enhances apoptotic cell ingestion by human activated macrophages (57, 67, 68). A well characterized recombinant fragment of human SP-D, comprising the homotrimeric neck and the carbohydrate recognition domain region, able to reach distal lung locations due to its smaller size and higher resistance to proteases and collagenases compared to the full size SP-D, appear to contribute to significant inhibition of infectivity and replication of SARS-CoV-2 present in the clinical samples derived from patients with asymptomatic, symptomatic, and severe COVID-19 (69). The ability of SP-D and recombinant fragment of human SP-D to bind SARS-CoV-2 S-protein suggest that they are capable of dampening the “cytokine storm” by rapid clearance of the virus-infected cells and strengthening the lung capacity by restoring homeostasis. Both, SP-A and SP-D have antiviral and immunomodulatory properties against SARS-CoV-2 (70, 71). SP-D participates in pulmonary viral neutralization, agglutination and clearance; reduction of the inflammatory response upon infection; enhancement of dendritic cell phagocytosis; enhancement of macrophage-mediated pathogen killing, inflammatory cytokine modulation and chemotaxis; modulation of intrapulmonary T-cell response; promotion of clearance of apoptotic cells to prevent necrosis and inflammation; and binding of neutrophil/eosinophil extracellular traps preventing degranulation and modulating cytokine production (70). SP-D recognizes SARS-CoV-2 S-protein, and recombinant SP-D bound to S-protein has been shown to inhibit interaction of the S-protein with ACE2 receptors (71). The importance of SP-A and SP-D for lung protection against viral infections has been demonstrated by the increased susceptibility of SP-A and SP-D knockout mice to influenza A and respiratory syncytial virus infections and viral-mediated inflammation (70). SP-D binds the heavily glycosylated SARS-CoV-2 S-protein (68); and SP-D levels, found to be elevated in severe SARS-CoV-2-related pneumonia, *via* leakage from the damaged lung into blood, may be used as biomarkers of clinical course, follow-up, determination of disease severity, and possibly future treatments for COVID-19 disease (72–76).

SP-A and SP-D bind to different surfactant protein receptors such as the complex CD91 (Low-density lipoprotein receptor-related protein 1, LRP1)-calreticulin (CRT) and signal inhibitory regulatory protein α (SIRPα) (**Figure 3**). Both (a) SP-A or SP-D bound to a virus or apoptotic cell through its lectin domain, and (b) apoptotic cells opsonized with C1q or MBL, bind to the CD91-CRT receptor through the collagen-like domain to activate immune cells and facilitate removal (**Figure 3**); while blockage of CD91 reduces apoptotic cell macrophage uptake (56, 77–80). SP-A and SP-D bind to toll-like receptors that recognize pattern-associated molecular patterns bound to RNA from viruses, and lung collectins and MBL cause (a) virion agglutination, inhibition of infectivity and dissemination, and (b) subsequent alveolar macrophage and neutrophil-mediated removal (56–58). In a normal environment, SP-A and SP-D fulfil an anti-inflammatory role mediated by the binding of their C-lectin domain to the SIRPα receptor (79).

The SIRPα/CD47 axis has emerged as an important innate immune checkpoint that enables cancer cells and virus-infected cells escape from macrophage phagocytosis (81–83). SARS-CoV-2-infected cells upregulate the CD47 “don’t eat me” signal, that could be used as potential biomarker of severe COVID-19 disease (84), slowing the phagocytic uptake of dying and viable cells (82). Immune inhibitory receptors like SIRPα become upregulated during viral infection as a feedback mechanism to prevent immune overactivation. During severe SARS-CoV-2 infection inflammatory cell immunosuppression and dysregulation with the resulting upregulation of inhibitory immune receptors like SIRPα, lead to immune exhaustion, suppression of innate and adaptive immune responses, and delayed interferon response with inefficient viral clearance, which could be directly induced by the virus as an evasion mechanism (85).

**Figure 4:**
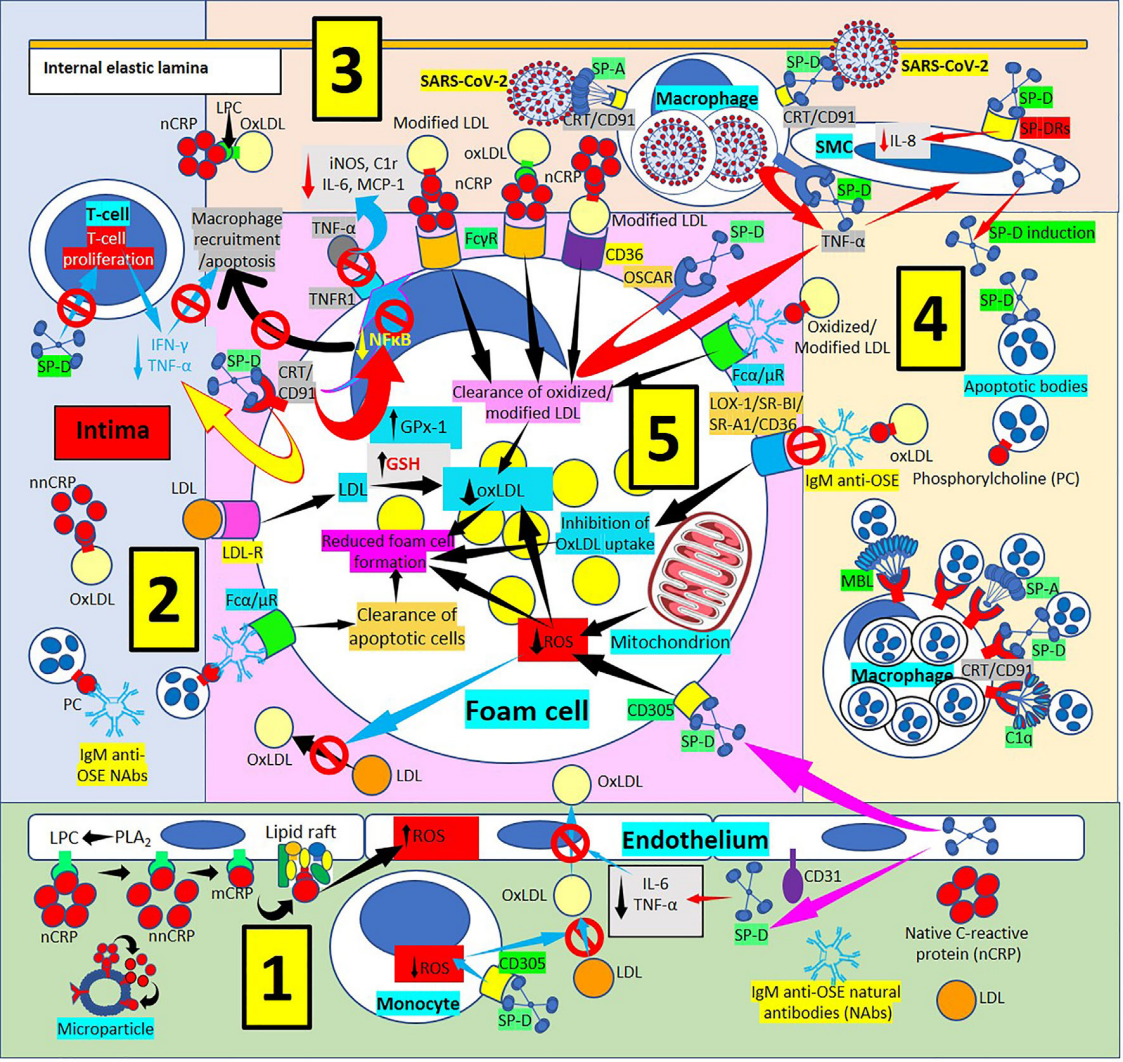
Surfactant protein innate immunoprotective responses in atherosclerotic lesions. [1] SP-D can be synthesized by endothelial cells and released into plasma and arterial intima, and circulatory SP-D inhibits circulatory IL-6 and tumor necrosis factor-α (TNF-α) and the transport of oxidized low-density lipoprotein (OxLDL) from the artery lumen to the subendothelial space; SP-D bound to CD305 (leukocyte-associated immunoglobulin-like receptor 1, LAIR1) reduces generation of reactive oxygen species (ROS) in foam cells (right and center in 1, and lower right in 5). SP-D bound to CD305 reduces ROS generation in circulating monocytes, and reduced ROS impedes oxidation of LDL (center in 1). Circulating native C-reactive protein (nCRP) bound to cellular or microparticle lysophosphatidylcholine (LPC) generated by phospholipase A_2_ (PLA_2_), or to phosphorylcholine (PC) can be dissociated to monomeric CRP (mCRP) with intermediate formation of non-native CRP (nnCRP), and mCRP can bind lipid rafts and increase ROS formation (left in 1). [2] SP-D reduces T-cell proliferation and interferon-γ (IFN-γ) and TNF-α release (top in 2). [3] [SP-D and SP-A facilitate intimal severe acute respiratory syndrome coronavirus-2 (SARS-CoV-2) virus removal by macrophages through CRT/CD91Rs (center in 3). SP-D binding to osteoclast-associated immunoglobulin-like receptor (OSCAR) in macrophages and foam cells enhances TNF-α release that stimulates SP-D production by smooth muscle (SMC) cells. SP-D also lowers SMC IL-8 (right in 3). [4] Pattern recognition proteins surfactant protein (SP)-A, SP-D, mannose binding lectin (MBL) and complement component 1q (C1q) facilitate macrophage uptake of apoptotic cells in atherosclerotic plaques through calreticulin (CRT)/CD91 receptors (Rs) (bottom in 4). [5] Immunoglobulin M (IgM) anti-oxidation-specific natural antibodies (OSE NAbs) in the arterial intima facilitate macrophage/foam cell uptake of apoptotic cells through Fcα/μRs (lower left in 5). Low-density lipoproteins (LDL) are taken by macrophages/foam cells through LDLRs (center in 5). Reduced foam cell ROS disallows LDL oxidation in the intima (lower left in 5). SP-D binding of CRT/CD91Rs leads to reduced inflammation characterized by reduced secretion of TNF-α in macrophages/foam cells; reduced nuclear factor κB (NFκB) activation and reduced macrophage recruitment and apoptosis; reduced expression of TNF-α receptor 1 (TNFR1) with concomitant reduction of foam cell TNF-α binding and reduced inducible nitric oxide synthase (iNOS), complement C1r protein, IL-6 and monocyte chemoattractant protein-1 (MCP-1) (upper left in 5). Native CRP (nCRP) facilitates clearance of oxidized/modified LDL through FcγRs and CD36 (upper right in 5); and IgM anti-OSE helps with clearance of oxidized/modified LDL through Fcα/μRs (upper right in 5), and by inhibiting their uptake through scavenger receptors lectin-like oxidized low-density lipoprotein (LDL) receptor-1 (LOX-1), scavenger receptor class B type 1 (SR-BI), scavenger receptor A1 (SR-A1) and CD36 (right in 5); all leading to reduced foam cell formation.

The most prevalent SP-D structure, the dodecameric conformation, seems to possess anti-inflammatory properties, while the trimeric or monomeric isoforms, are believed to have pro-inflammatory properties, when SP-D is in an inflammatory or more acidic environment (86–91). Surfactant is mainly produced in the lung, but surfactant mRNA and protein have also been identified in heart, arteries, kidney, and other organs (56, 86, 92) (**Figures 3**, **4**); and SP-A and SP-D seem to modulate the immune response by preventing excessive inflammation, as found in the cytokine storm caused by COVID-19, that can potentially damage the lungs and impair gas exchange (55). Pulmonary SP-D production can be enhanced by tumor necrosis factor-α (TNF-α)-mediated induction of IL-13 (90) (**Figure 3**). LRP1 (CD91)-bound SP-D may be antiatherogenic by decreasing TNF receptor-1 expression, attenuating nuclear factor-kB activation, limiting monocyte recruitment into atherosclerotic plaques, preventing macrophage apoptosis, and enhancing dying cell digestion limiting necrotic core formation (77, 93–95). C1q and MBL bind apoptotic cells that can be engulfed by macrophages following binding of their collagenous tails to CRT (cC1qR)/CD91 surface receptors (79). SP-A and SP-D interact with foreign organisms, like SARS-CoV-2, apoptotic cells or cell debris through LRP1/CRT-mediated macrophage phagocytosis and modulation of the inflammatory response in lungs and atherosclerotic plaques (57, 68, 95). The ability of CRT/CD91 to scavenge microbial membrane lipoproteins and to digest apoptotic cells favors the control of local inflammation by preventing an overwhelming inflammatory response causing further injury, as found during SARS-CoV-2 infection-associated hyperinflammation and cytokine storm (70, 96).

**Figure 5:**
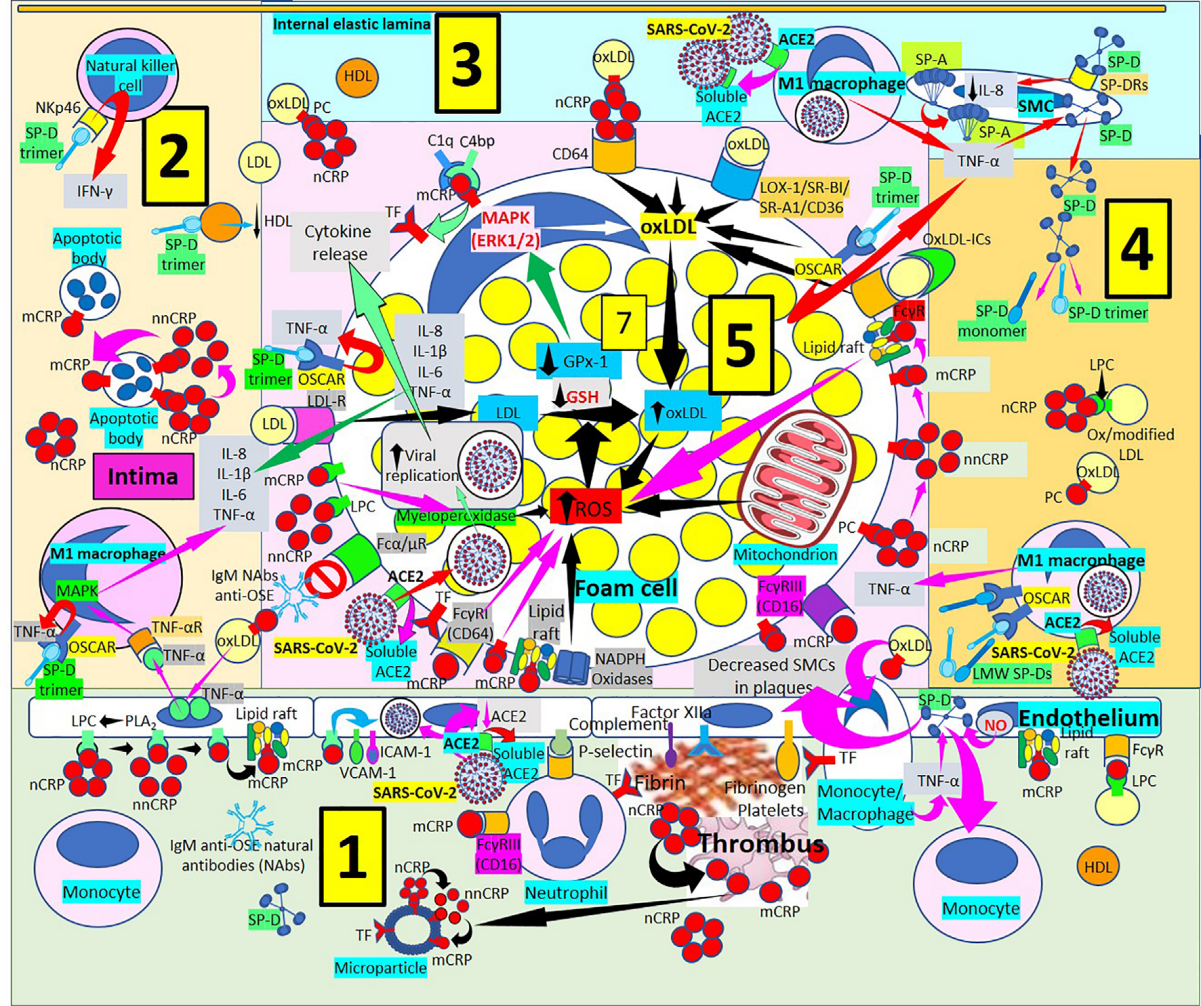
Surfactant proteins in atherogenesis. [1] Monomeric C-reactive protein (mCRP) bound to Fcγ receptors (Rs) and lipid rafts is proinflammatory and induces endothelial nitric oxide (NO) (right in 1). NO and monocyte/macrophage tumor necrosis factor-α (TNF-α) increase surfactant protein D (SP-D). SP-D and oxidized LDL (oxLDL) attract monocytes into the intima and SP-D decreases smooth muscle cells (SMCs) in plaques (right in 1). Monocytes/macrophages express tissue factor (TF), and endothelial-bound procoagulants (factor XIIa, fibrinogen) and complement participate in fibrin and thrombus formation further generating mCRP and microparticles (center right in 1). Monomeric CRP *via* FcγRIII promotes neutrophil recruitment into the plaques *via* endothelial P-selectin (center in 1). Circulating native CRP (nCRP) bound to cellular lysophosphatidylcholine (LPC) generated by phospholipase A2 (PLA2) can be dissociated to mCRP with intermediate formation of non-native CRP (nnCRP); mCRP binds lipid rafts and cellular LPC and activate adhesion molecules intercellular adhesion molecule-1 (ICAM-1) and vascular cell adhesion molecule-1 (VCAM-1) (left in 1). Severe acute respiratory syndrome coronavirus-2 (SARS-CoV-2) can invade endothelial cells through angiotensin converting enzyme 2 (ACE2), release soluble ACE2 and reduce endothelial ACE2 (center left in 1). [2] OxLDL-induced endothelial TNF-α release stimulates TNF-α receptor (R)-mediated mitogen-activated protein kinase (MAPK) activation and proinflammatory cytokine release. SP-D trimers stimulate osteoclast-associated receptor (OSCAR)-mediated TNF-α release. Dissociation of nCRP to mCRP passing through nnCRP occurs in intimal apoptotic cells. SP-D trimers lower high-density lipoproteins (HDL), and bind NKp46 to activate natural killer (NK) cells releasing interferon-γ (IFN-γ). [3] Macrophages invaded by SARS-CoV-2 facilitate soluble ACE2 release that could neutralize more viruses. SP-D trimer OSCAR-mediated TNF-α release from foam cells (center right in 3 and upper right in 5) and macrophages stimulates SP-D synthesis and release from smooth muscle cells (SMCs) allowing further development of trimers and monomers under inflammation. SP-A is synthesized in SMCs and SP-D bound to SP-DRs lower IL-8 (right in 3). [4] Low molecular weight (LMW) SP-Ds (trimers, monomers) bind OSCAR in macrophages and release more TNF-α enhancing inflammation. [5] OxLDL uptake, increased ROS production and reduced antioxidant activity (glutathione peroxidase, GPx-1; glutathione, GSH) enhance foam cell formation (center in 5). SARS-CoV-2 can invade macrophages/foam cells through ACE2 and release soluble ACE2 (lower left in 5). Monomeric CRP through FcγRs and bound to phosphorylcholine (PC) and lipid rafts associated with nicotinamide adenine dinucleotide phosphate (NADPH) oxidases can increase reactive oxygen species (ROS) and TF (lower left and center lower left in 5). OxLDL bound to IgM natural antibodies (NAbs) can block Fcα/μ-mediated oxLDL uptake. CRP (most probably mCRP and/or nnCRP) can activate myeloperoxidase and generate ROS (lower left in 5). LDL is taken by LDL-Rs into the cell (center left in 5). LMW SP-Ds (trimers and/or monomers) bound to OSCAR can enhance TNF-α release, and foam cells also release further TNF-α, interleukin (IL)-8, IL-1ß and IL-6 contributing to inflammation (center left in 5). Complement component 1q (C1q) and C4b-binding protein (C4bp) compete for mCRP to adjust local complement activation/inhibition balance; mCRP enhances TF expression (upper left in 5). OxLDL is transported into foam cells through CD64 (bound to CRP); scavenger receptors lectin-like oxidized low-density lipoprotein (LDL) receptor-1 (LOX-1), scavenger receptor class B type 1 (SR-BI), scavenger receptor A1 (SR-A1) and CD36; and FcγRs (oxLDL immune complexes, oxLDL-ICs) (upper left, center and right in 5). Native CRP generates mCRP in foam cell membranes and lipid raft-bound mCRP enhances ROS (center right in 5).

SP-D and the SP-D-receptor, osteoclast-associated receptor (OSCAR), are found in atherosclerotic plaques (86, 97–101) (**Figures 4**, **5**), particularly in an inflammatory environment where TNF-α and oxidized low-density lipoprotein (oxLDL) induce SP-D and OSCAR expression, respectively (86, 97–104). SP-D promotes atherogenesis by 1) Lowering HDL levels, 2) Enhancing OSCAR-mediated macrophage TNFα production, 3) Inducing circulating monocytes and macrophage proliferation, 4) Enhancing foam cell accumulation and plaque formation and 5) Decreasing atherosclerotic plaque smooth muscle cell coverage (97, 99–101). In an inflammatory and oxidative environment, endothelial, smooth muscle and macrophage dodecameric SP-D production breaks apart into trimeric and monomeric isoforms (100–106); and in association with increased cytokine production in atherosclerosis, make this lesion more prone to SARS-CoV-2 infection, especially considering that advanced atherosclerotic lesions show increased ACE2 receptor expression (107, 108), the SARS-CoV-2 virus receptor. SARS-CoV-2 infection in the plaques contributes to diminished ACE2 regulation, exposing endothelial cells to angiotensin II in the absence of modulatory effects of angiotensin 1-7, worsening atherosclerosis (108).

In summary, SP-A and SP-D perform a dual role, anti-inflammatory and proinflammatory, depending upon the part of the molecule that binds the receptor (C-lectin domains or collagenous tail region) and the type of receptor bound (CRT/CD91 or signal inhibitory regulatory protein α, SIRPα). Furthermore, multimeric and trimeric/monomeric SP-D isoforms perform opposite functions during inflammation, promoting the idea of “good” and “bad” SP-D. Multimeric, mainly dodecameric or fuzzy form, will contribute to viral neutralization, agglutination and clearance, as well as removal of infected apoptotic cells, in lungs and atherosclerotic plaques. On the contrary, trimeric and monomeric SP-D isoforms generated during oxidative stress will enhance inflammation and disease progression in lungs, atherosclerotic lesions and other organs. Reduction in the generation of trimeric/monomeric forms will be most probably associated with lowering the severity of pulmonary damage and plaque size and progression (100, 101, 105). Deficiency of multimeric forms could be related to a proinflammatory environment with abnormal accumulation of apoptotic macrophages and foamy macrophages in lungs and plaques (100, 101, 105). In a normal environment, SP-A and SP-D fulfil an anti-inflammatory role mediated by the binding of their C-lectin domain to the SIRPα receptor. During acute inflammation of the lungs, a deficient apoptotic cell clearance is overcome by the recruitment of mononuclear phagocytes and following the binding of foreign particles (viruses or apoptotic bodies) alveolar macrophages initiate inflammation and host defense reactions (88, 109). SP-D and SP-A binding to alveolar macrophages during inflammation occurs *via* their C-lectin domain and subsequent binding to the CRT/CD91 receptor complex *via* their collagenous tail region (88, 109). Enhancement of apoptotic cell clearance during SARS-CoV-2 infection is paramount, especially when it has been shown that a dysfunctional efferocytosis in SARS-CoV-2-infected cell corpses suppresses macrophage anti-inflammation and efficient tissue repair allowing hyperinflammation and extensive tissue damage linked with COVID-19 (110).

COVID-19 disease originates in the lungs but affects multiple organs causing multiorgan damage in severe cases, and this tissue injury is primarily the result of hypercytokinemia and aggressive inflammation (111). The manifestations of severe COVID-19 such as the acute respiratory distress syndrome, sepsis and multiorgan failure have an established relationship with activation of the complement cascade (112). Collectins (SP-A, SP-D, and MBL) and C1q, which is related to the collectins by structure and function, except that its globular head lacks lectin activity, are members of the innate immune system that play key roles in the early recognition and removal of microorganisms, modulation of the inflammatory response, and clearance of apoptotic cells (80); and the entire collectin family (including C1q) works through a common receptor complex and are integral, organ-specific components of the clearance machinery (80).

## C-reactive Protein and the Coronavirus

### C-Reactive Protein and Inflammation

C-reactive protein (CRP) discovered in 1930 by Tillett and Francis Jr is an acute-phase pattern recognition protein (PRP) considered to be a sensitive biomarker of systemic inflammation, mainly produced by liver hepatocytes, secondary to cytokine stimulation (i.e., IL-6, IL-1β or TNF-α) (113–115) that increases 1,000-10,000-fold at sites of infection or inflammation within 24-72 hours (114, 116–126), from the 0.8 mg/L found in healthy young adult individuals (119). CRP is also synthesized in extrahepatic cells and tissues including smooth muscle cells of atherosclerotic lesions with active disease, foam cells, macrophages, alveolar macrophages, epithelial cells of the respiratory tract, lymphocytes, monocytes, and endothelial cells, among others (127–135).

CRP binds and aggregates oxidized low-density lipoprotein (ox-LDL) and enhances macrophage oxLDL uptake, leading to mitogen-activated protein kinase activation (136) and foam cell formation (137). CRP binds to many ligands (118–120, 138–141); phosphorylcholine (PC) being the most characterized, that starts damaged cell recognition and phagocytosis (118, 119, 121) and is responsible for binding to microbial capsular polysaccharide, oxLDL and apoptotic cells, but not either native LDL or viable cells not exposing the PC moieties (118, 119, 133, 138, 139). CRP amino acids Phe^66^ and Glu^81^ promote the binding of CRP to PC (138, 142, 143), and the opposite face of native (n)CRP binds complement Cq1 and Fcγ receptors, whose binding sites are overlapping (119, 138).

**Figure 6:**
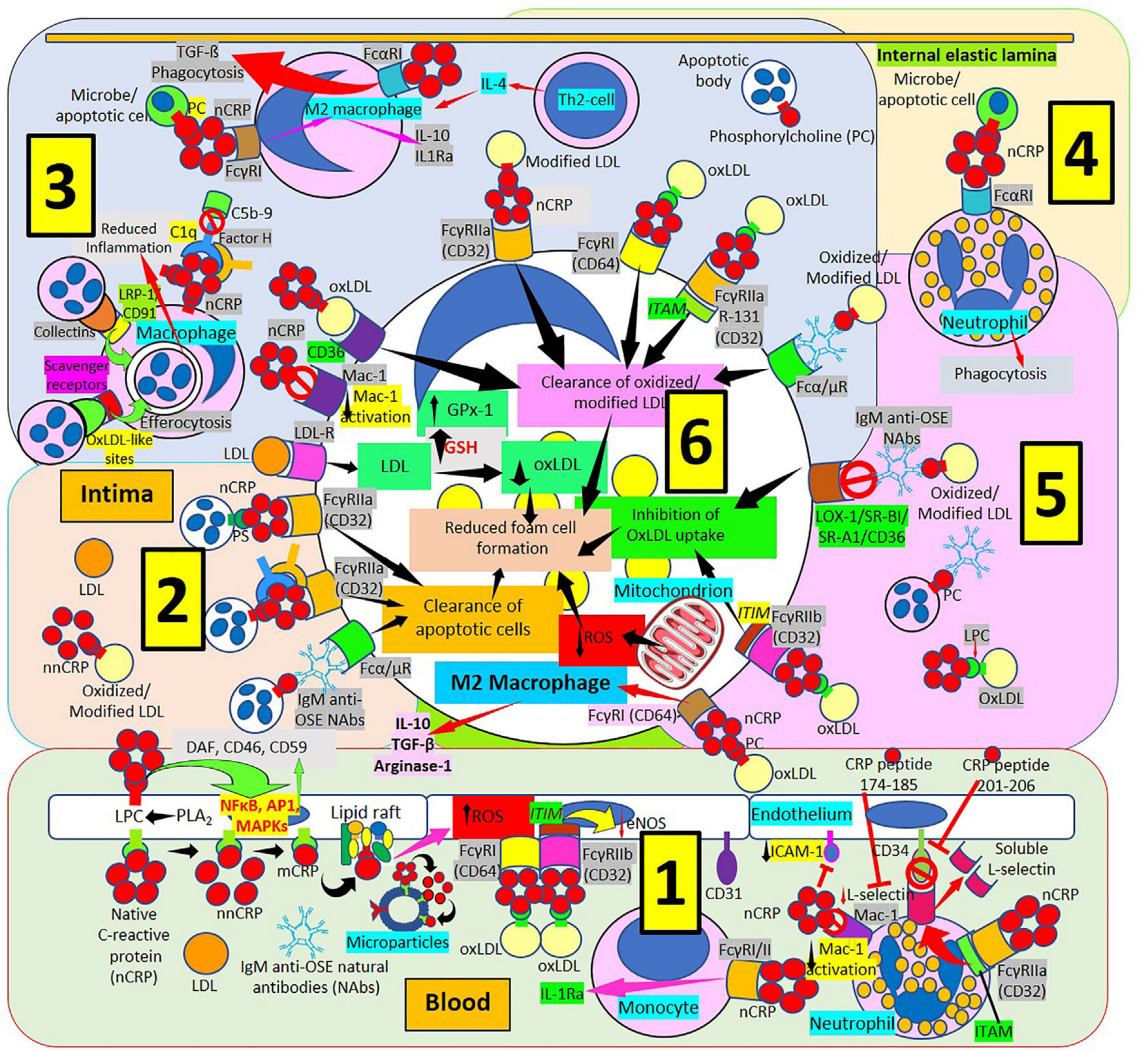
C-reactive protein innate immunoprotective responses in atherosclerotic lesions. [1] Native pentameric C-reactive protein (nCRP) and CRP peptides prevent neutrophil adhesion by downregulating L-selectin expression through activation of Fcγ receptor (R) IIa and immunoreceptor tyrosine-based activation motif (ITAM), and by inducing L-selectin shedding; nCRP downregulates CD11b/CD18 (Mac-1) receptor activation and endothelial intercellular adhesion molecule-1 (ICAM-1) expression (right in 1). Native pentameric CRP induces FcγR-mediated interleukin (IL)-1 receptor antagonist (Ra) expression in mononuclear cells (middle in 1). CRP bound to oxLDL can reduce endothelial nitric oxide synthase (eNOS) through FcγRs and immunoreceptor tyrosine-based inhibitory motif (ITIM) activation (middle in 1). Circulating nCRP bound to cellular or microparticle lysophosphatidylcholine (LPC) generated by phospholipase A_2_ (PLA_2_), or to phosphorylcholine (PC) can be dissociated to monomeric CRP (mCRP) with intermediate formation of non-native CRP (nnCRP), and mCRP can bind lipid rafts and increase reactive oxygen species (ROS) formation (left in 1). Native CRP can prevent cell damage by enhancing membrane-bound expression of complement regulators decay-accelerating factor (DAF), membrane cofactor protein (CD46) and CD59 mediated by transcription factor (nuclear factor kappa B, NFκB; activator protein 1, AP-1; mitogen-activated protein kinases, MAPKs) activation. [2] The clearance of apoptotic cells is facilitated by immunoglobulin M anti-oxidation specific epitopes natural antibodies (IgM anti-OSE NAbs) and Fcα/μRs; nCRP-PC, complement 1q (C1q), factor H and FcγRIIa; and nCRP-phosphatidylserine (PS) and FcγRIIa, respectively. Low-density lipoproteins (LDL) uptake occurs *via* LDL-Rs. [3] Native CRP downregulates Mac-1 activation. Native CRP, C1q and factor H; collectins bound to low density lipoprotein receptor-related protein (LRP)-1/CD91; and oxLDL-like sites bound to scavenger receptors facilitate efferocytosis. CRP bound to PC and FcγRI induces M2 macrophage differentiation that reduces inflammation through IL-10 and IL-1R antagonist (IL1Ra) secretion. Th2 T-lymphocytes release IL-4 further inducing M2 macrophages. CRP binds FcαRI facilitating transforming growth factor-ß (TGF-ß) release and phagocytosis. FcγRs CD32 and CD64, scavenger receptor CD36, FcγRIIa R131 and Fcα/μR facilitate clearance of oxidized/modified LDL. [4] FcαRI mediates nCRP neutrophil stimulation of phagocytosis. [5] Immunoglobulin M (IgM) anti oxidation-specific epitopes (OSE) blocks scavenger receptors lectin-like oxidized low-density lipoprotein (LDL) receptor-1 (LOX-1), scavenger receptor class B type 1 (SR-BI), scavenger receptor A1 (SR-A1) and CD36 inhibiting oxLDL uptake. Native CRP through CD32 and immunoreceptor tyrosine-based inhibitory motif (ITIM) inhibits oxLDL uptake; and through CD64 binding induces M2 macrophage differentiation that enhances release of IL-10, TGF-ß and arginase 1. [6] Clearance of oxidized/modified LDL, inhibition of oxLDL uptake, clearance of apoptotic cells, reduced reactive oxygen species (ROS) and reduced oxLDL formation associated with increased glutathione (GSH) and glutathione peroxidase-1 (GPx-1) activity contribute to reduced foam cell formation.

Pentameric nCRP, recognized as an antibody ancestor (144), is constituted by five identical monomers (homopentamer), known as nCRP. CRP can be identified in three different forms: 1) nCRP, 2) Non-native pentameric CRP (nnCRP), and 3) Monomeric CRP (mCRP) (118–126). Native nCRP can irreversibly dissociate at sites of inflammation/infection into five separate mCRP monomers, each containing 206 amino acids with a molecular mass of about 23 kDa (118–123, 130, 133, 145–161). The different CRP isoforms may explain their protective and destructive effects, with nCRP being predominantly anti-inflammatory inducing Th2/M2 responses, while mCRP being mainly proinflammatory inducing Th1/M1 responses (146, 161–165). Pentameric nCRP bound to PC acts *via* membrane glycoprotein Fcγ receptor (R) I to promote M2 macrophage differentiation dependent on IL-13 (146). Pentameric nCRP maintains an anti-inflammatory response by: a) Enhancing phagocytosis and apoptotic cell clearance, b) Protecting against cell lysis through factor H recruitment and preventing assembly of the complement membrane attack complex, c) Preventing cell damage by enhancing membrane-bound expression of complement regulators decay-accelerating factor, membrane cofactor protein, and CD59, d) inhibiting nitric oxide production, and e) Inducing mononuclear cell interleukin-1 receptor antagonist maintaining an anti-inflammatory cytokine profile (119, 161, 166, 167). All these effects require the presence of C1q, indicating a pivotal role for the early complement component(s) in the anti-inflammatory effects of CRP (167) (**Figure 6**). Pentameric nCRP and CRP peptides 77-82, 174–185 and 201–206 can limit the inflammatory response resolving inflammation by reducing neutrophil endothelial adhesion and tissue migration (159, 162, 168)

**Figure 7:**
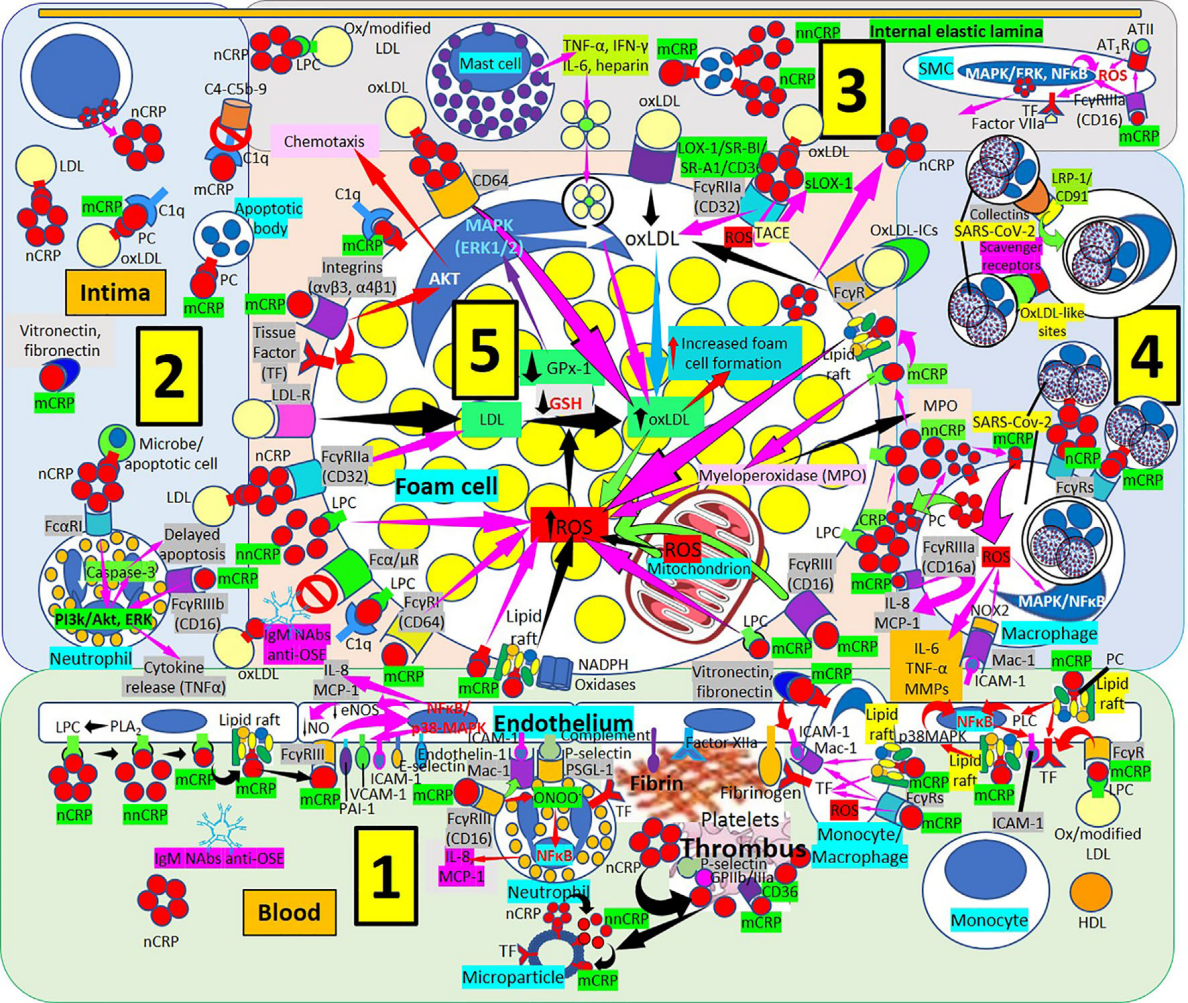
Monomeric C-reactive protein in atherogenesis. [1] From right to left. Monomeric C-reactive protein (mCRP) bound to oxidized/modified (ox/modified) low density lipoprotein (LDL) through lysophosphatidylcholine (LPC) binds Fcγ receptors (Rs) upregulates endothelial tissue factor (TF) (right in 1). Monomeric CRP bound to phosphorylcholine (PC) in lipid rafts activate intracellular signaling cascades (phospholipase C, PLC; p38 mitogen activating protein kinases, MAPK; nuclear factor κB, NFκB) enhancing endothelial intercellular adhesion molecule-1 (ICAM-1) (right in 1). Monocytes/macrophages adhere to endothelium following mCRP-mediated ICAM-1 and CD11b/CD18 (Mac-1) receptor upregulation; mCRP in monocytes/macrophages upregulate TF and Mac-1 and adhere to vitronectin/fibronectin in the intima (right in 1). TF, factor XIIa, fibrinogen and complement participate in thrombus formation and platelets generate further mCRP from nCRP. Interaction among mCRP, glycoprotein (Gp) IIb/IIIa, P-selectin and CD36 participate in inflammation and thrombus formation. Endothelial and platelet microparticles generate mCRP from nCRP and nonnative CRP (nnCRP) (center right in 1). Monomeric CRP facilitates endothelial neutrophil adherence *via* FcγRIII and Mac-1 upregulation; neutrophil Mac-1 and P-selectin glycoprotein ligand (PSGL)-1 attach to ICAM-1 and P-selectin, respectively; mCRP- FcγRIII enhance peroxinitrite (ONOO^-^) generation, NFκB activation and IL-8 and monocyte chemoattractant protein-1 (MCP-1) release (center in 1). Endothelial phospholipase A_2_ (PLA_2_) generates LPC facilitating monomerization of CRP that binds lipid rafts and FcγRIII and activates NFκB/p38MAPK, upregulating plasminogen activator inhibitor (PAI)-1, vascular cell adhesion molecule (VCAM)-1, E-selectin and endothelin-1; downregulating endothelial nitric oxide synthase (eNOS) and nitric oxide (NO); and enhancing IL-8 and MCP-1 release (left and center left in 1). [2] CRP *via* FcαRI and mCRP *via* FcγRIIIb can activate phosphatidylinositol 3-kinases/protein kinase B and extracellular signal-regulated kinases (PI3k/AKT, ERK) signaling pathways in intimal neutrophils and enhance tumor necrosis factor-α (TNF-α) release and delay apoptosis. T-cells can produce CRP enhancing intimal CRP. [3] Mast cells contribute to oxLDL macrophage uptake through TNF-α, interferon-γ (IFN-γ), IL-6 and heparin secretion. CRP can be synthesized by smooth muscle cells (SMC) and foam cells, and FcγRIIIa-bound mCRP in SMCs induce ROS and upregulate TF enhancing thrombus formation through factor VII activation; mCRP can upregulate angiotensin (AT)_1_R and ATII-mediated ROS production. [4] LRP-1/CD91 and scavenger receptors facilitate collectin- and oxLDL-like site-mediated severe acute respiratory syndrome coronavirus-2 (SARS-CoV-2) uptake, respectively, and FcγRs can facilitate nCRP and mCRP-mediated SARS-CoV-2 macrophage phagocytosis; mCRP and ICAM-1 can mediate ROS formation and subsequent release of IL-8, MCP-1, IL-6, TNF-α and matrix metalloproteinases (MMPs). [5] Increased ROS formation, reduced glutathione (GSH) and glutathione peroxidase (GPx)-1 activity increase foam cell formation sustaining inflammation in the plaque (center in 5). OxLDL bound to IgM natural antibodies (NAbs) can block Fcα/μ-mediated oxLDL uptake. Monomeric CRP bound to lipid rafts, LPC, PC or FcγRs and LPC-bound nonnative CRP (nnCRP) could enhance ROS formation; and LPC bound mCRP activate C1q (lower left in 5). CRP/CD32 and LDL-R facilitate macrophage LDL uptake (center left in 5). CRP bound to CD64 enhance oxLDL uptake and foam cell formation; mCRP contributes to intimal inflammation, integrin-mediated TF upregulation and chemotaxis and C1q activation (upper left in 5). Scavenger receptors lectin-like oxidized low-density lipoprotein (LDL) receptor-1 (LOX-1), scavenger receptor class B type 1 (SR-BI), scavenger receptor A1 (SR-A1), and CD36,facilitate oxLDL macrophage uptake; and FcγRs CRP-bound oxLDL and oxLDL immune complexes (oxLDL-ICs) (upper center and upper right in 5). LPC membrane-bound nCRP dissociates into mCRP with intermediate nnCRP formation, and mCRP can induce myeloperoxidase (MPO) synthesis and release; LPC- and lipid raft-bound mCRP can enhance ROS formation (right in 5). Monomeric CRP bound to CD16 or LPC can enhance foam cell ROS (right in 5).

Pentameric nCRP binds to FcγRIIa (CD32) and FcγRI (CD64) on polymorphonuclear leukocytes and monocytes/macrophages although the major receptor for nCRP on phagocytic cells is FcγRIIa (169–178). Native nCRP binds FcαRI/CD89 on neutrophils and macrophages, and, in neutrophils, nCRP induces FcαRI surface expression, phagocytosis and TNF-α secretion (179). The binding of nCRP to both stimulatory receptors, FcγRI and FcγRIIa, increases phagocytosis and the release of inflammatory cytokines; and to the inhibitory receptor, FcγRIIb, by blocking activating signals, allows nCRP to maintain an equilibrium leading to a predominant anti-inflammatory effect (114, 115). CRP-enhanced opsonization of apoptotic cells and subsequent phagocytosis by macrophages, mediated through FcγR-CRP-binding, impedes inflammation caused by necrotic and apoptotic cells (180) (**Figures 6, 7**).

Monomeric mCRP is considered to be proinflammatory because a) Stimulates leukocyte chemotaxis and recruitment to areas of inflammation, b) delays apoptosis; c) Increases interleukin-8 and monocyte chemoattractant protein-1 production; and d) Induces nitric oxide production (161, 181). Ligand-bound nCRP can dissociate into mCRP which modulates complement-mediated inflammation and activates neutrophils and monocytes (133). When mCRP is in a fluid phase, in ligand-free state, inhibits C1q binding to other complement activators; but when mCRP is immobilized on the cell surface either alone or bound to ox-LDL or enzyme-modified (E)-LDL, it binds and activates complement C1q, leading to the turnover of C3, to a great extent bypassing the more inflammatory and destructive terminal sequence by recruitment of Factor H (157) (**Figures 6**, **7**).

The mCRP pro-inflammatory effects suggest that mCRP is proatherogenic. Indeed, mCRP, but not nCRP, a) Induces reactive oxygen species monocyte/macrophage production and facilitates macrophage uptake of necrotic cells (158); and b) Causes neutrophil activation, firm cell adhesion (159, 162) and repression of neutrophil apoptosis, facilitating neutrophil survival, and amplifying the acute inflammatory response; both contributing to atherosclerotic plaque formation and plaque rupture or destabilization (150, 152). Dissociation of nCRP on activated cell membranes occurs *via* phospholipase A2-mediated lysophosphatidylcholine generation (149, 150) (**Figure 7**). The mCRP isoform uses the low-affinity immune complex binding immunoglobulin G (IgG) receptor FcγRIIIb (CD16b) on neutrophils and the FcγRIIIa (CD16a) on monocytes (161). The link of mCRP with FcγRIII (CD16) in human neutrophils and endothelial cells (177, 178, 182–184), other receptors of the FcγR family (185), integrins αvβ3 and α4β1 (186), fibronectin, vitronectin, as well as lipid rafts microdomains on cell membranes are essential for mCRP-mediated cellular signaling and its proinflammatory effects (187). Monomeric mCRP promotes: 1) Increased arterial endothelial monocyte chemoattractant protein-1 and interleukin-8 (IL-8) production and enhanced intercellular adhesion molecule-1, E-selectin, and vascular adhesion molecule-1 expression (184); and 2) Increased IL-8 production by human neutrophils through peroxynitrite (ONOO^-^)-mediated activation of nuclear factor-κB and activator protein 1 (183).

Non-native CRP (nnCRP) generated during membrane-bound nCRP dissociation (187) into subunits retaining some of the native conformation before fully dissociating into mCRP, seems to be, as mCRP, proinflammatory, allowing more effective CRP function regulation, and enhancing activation of the classical complement pathway (160, 161, 181). Pentameric nnCRP could also be atheroprotective since nCRP and nnCRP bind atherogenic LDL and reduce foam cell formation and atherogenic LDL-associated inflammation (188, 189). Pentameric nnCRP has a more relaxed structure when compared to nCRP, exposing neoepitopes indispensable for both immune and complement activation. The dissociation/relaxation of nCRP into nnCRP occurs on necrotic, apoptotic, and ischemic cells, membranes of activated platelets, monocytes, and endothelial cells, and on the surface of microparticles, *via* PC binding. Pentameric nCRP does not possess intrinsic pro-inflammatory properties, while nnCRP and mCRP do (181). Both nnCRP and mCRP can activate platelets, leukocytes, endothelial cells, and complement (181). Pentameric nnCRP seems the dominant proinflammatory isoform (181), and by exposing amino acids 199-206, shows mCRP-like antigenicity being recognized by antibodies to mCRP, and enhances complement fixation (190). Furthermore, a form of CRP, most probably mCRP, enhances vascular smooth muscle cell tissue factor expression *via* p44/42 mitogen-activated protein kinase and increased production of reactive oxygen species, promoting thrombosis in atherosclerotic plaques (191, 192). Interactions of mCRP with fibronectin (193), vitronectin, and other proteins, could be part of an available system to eliminate excess mCRP and/or scavenge altered, damaged and denatured proteins limiting inflammation in the arterial wall (120).

The interaction between mCRP, but not nCRP, and fibronectin occurs in residues 35-47 of mCRP that becomes exposed after nCRP dissociation. Monomeric mCRP can enhance monocyte fibronectin adhesion and upregulate endothelial cell adhesion molecule expression (**Figure 7**). Two major FcγRs, Fcγ-RI (CD64), and Fcγ-RIII (CD16) were identified as the major FcγRs on human monocytes to promote inflammation independently of lipid raft signaling. Enriched lipid raft microdomains are the major surface sensors for mCRP on the apical membranes of endothelial cells (149). Endothelial cell apical mCRP being present in circulation for an extended period of time as a consequence of endothelial damage, enhanced conversion from nCRP on microparticles or activated platelets which can facilitate endothelial cell activation through phospholipase C, p38 and nuclear factor-κB signaling pathway induction, contribute to mCRP-mediated chronic vascular inflammation. Phospholipase A2–mediated lysophosphatidylcholine generation in endothelial cell apical membranes could enable nCRP dissociation in circulating inflammatory cells generating mCRP and triggering leukocyte-endothelium interaction further enhancing inflammation (149, 150). The mCRP isoform, unlike nCRP, has a stimulatory effect on platelets, facilitates thrombus growth through platelet stimulation, and is the more potent reagent, both increasing monocyte activation and production of reactive oxygen species, which could be generated through myeloperoxidase-mediated respiratory burst and raft-associated reduced nicotinamide adenine dinucleotide phosphate (NADPH)-oxidase during oxLDL-mediated foam cell formation (194). Thrombus formation and the subsequent activation of the coagulation cascade with final generation of fibrin is facilitated by the mCRP-mediated enhancement of tissue factor on the endothelial cell surface, platelet aggregation and thrombus growth (153, 154). CRP seems to play a major role in inflammation and host responses to infection by its effects on the complement pathway, apoptosis, phagocytosis, nitric oxide release, release of proatherosclerotic factors like soluble lectin-like oxidized low-density lipoprotein receptor-1 (195, 196) and production of cytokines, particularly interleukin-6 and tumor necrosis factor-α.

### C-Reactive Protein, Coronaviruses and SARS-CoV-2

Increased CRP levels have been described in patients infected with the most virulent types of influenza A virus, and the outcome of human influenza disease has been related to increased CRP production, with the highest levels of CRP corresponding to the more severe symptoms including mortality (197–201). Similarly, high levels of CRP were found in patients with severe COVID‐19 disease showing increased organ damage, worse outcome, and increased mortality (202–206). The hyperinflammation associated with COVID-19 infection in children and adults (207–210) affects all organs and tissues including atherosclerotic lesions (**Figure 7**) and is always associated with very high levels of CRP, that enhance and amplify the inflammatory and prothrombotic microenvironment, most certainly mediated by increased generation of mCRP, which could also contribute to plaque instability through the demonstrated CRP-induced expression of matrix metalloproteinases 1, 2, and 9 (154). Considering the pathophysiology of COVID-19 infection and its complications, it is appealing to propose that membrane-associated monomeric CRP isoform may play a role, particularly promoting pro-inflammatory and procoagulant effects (205, 206). Viruses utilize host cells to reproduce and viral infections eventually result in cell death, caused by cell’s surface membrane alterations, apoptosis, and cell lysis (211, 212). Therefore, the enhancement of apoptosis-dependent phagocytosis of virus-infected cells is a fundamental approach to eliminate the virus without increasing inflammation, since apoptosis is considered a silent mode of cell death (213). CRP, in the presence of calcium, binds to phosphorylcholine (PC) residues present in pneumococcal C-polysaccharide of streptococcus pneumoniae and PC exposed on damaged and apoptotic cells (138, 139). CRP can block the attachment of bacteria expressing cell-surface PC to host cells (214), and at the same time facilitate complement-mediated bacteriolysis and complement/factor H-mediated macrophage phagocytosis. The proinflammatory effects of mCRP in the alveoli may be inhibited by PC in lung surfactant, which is abundant in the terminal airway where the major lipid component is dipalmitoyl phosphatidylcholine that has a polar head group consisting of PC (214). The collectins, surfactant proteins A and D, enhance the antimicrobial activity of alveolar macrophages (214), and the phospholipid component of surfactant has both immune-enhancing and inhibitory effects (214, 215). The CRP provided by the increased plasma levels secondary to elevated CRP hepatocyte synthesis and the local production by epithelial cells of the human respiratory tract (132) can facilitate the antimicrobial activity of bacteria complicating any RNA coronavirus infection such as COVID-19. This occurs specifically by targeting organisms that express cell-surface PC; since nCRP can bind the bacterial PC moieties and inhibit the platelet activating factor receptor-mediated microbe epithelial cell adherence and invasion (214). Since the concentration of the phospholipid component of surfactant is estimated to be 5 mg/ml, surfactant may not always have a protective function, but may inactivate the innate CRP-mediated defense allowing PC-expressing bacteria to adhere to and invade alveolar epithelial cells (214). The highly elevated CRP levels during infection and inflammation especially in association with hyperinflammation during coronavirus infection, may facilitate the neutralization of bacterial PC impeding attachment and invasion of alveolar epithelial cells. The marked elevation of CRP levels during the hyperinflammation found in severe cases of COVID-19 infection, however, typically has a detrimental outcome. The explanation may relate to the concomitant generation of increased amounts of mCRP.

## IgM Pattern Recognition Protein, Innate Immune Responses and the Coronavirus

**Figure 8:**
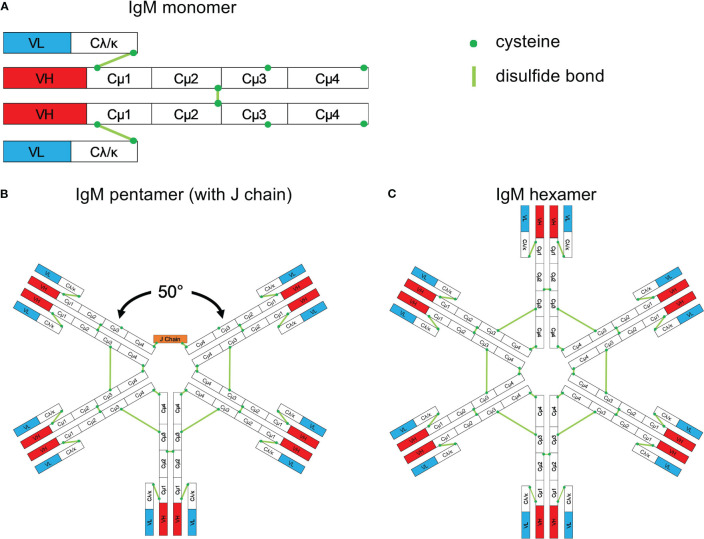
Schematic structure of IgM. **(A)** Monomeric IgM is composed of two heavy (μ) and two light (λ/κ) chains. Each heavy or light chain contains one variable region (VH or VL) and one constant region (Cμ1-4 and Cλ/κ). **(B)** Pentameric IgM contains five monomers and one J chain; disulfide bonds between each monomer form the pentamer; the structure shown in **(B)** is based upon the recent electron microscopy image presented by Hiramoto et al. (221). There is a 50° gap where the J chain resides. **(C)** The IgM hexamer contains six monomers and resembles a hexagon. The J chain is generally absent in hexamers. With permission from Gong and Ruprecht, (220).

Immunoglobulin M (IgM) antibodies, first identified in the 1930s as high molecular weight antibodies (216–218), account for the major integrant of the natural antibodies and is also the first antibody type produced during a primary antibody response (219). IgM was identified immediately following the discovery of CRP, another major component of innate immunity (113). IgM is the first responder to foreign invaders including the viruses that have caused major pandemics, such as the current COVID-19 virus. IgM, considered an ancient antiviral weapon, exists in all vertebrates (220). Its monomeric form (**Figure 8**) is expressed on B cells as the B-cell antigen receptor (220). IgM is secreted mainly as a pentameric molecule (**Figure 8**) (220, 221) containing a joining chain (J chain), and in humans has a high serum concentration (1.5 mg/ml).

Following the initial characterization of the IgM antibody (219), much is now known regarding its structure, production, and function. It is globally recognized that IgM provides a first line of defense during microbial infections, before a high affinity IgG adaptive immune response develops imparting lasting immunity and immunological memory. Detection of an IgM immune response is recognized as a measure of being exposed to infectious pathogens, however, the role of IgM antibodies in many microbial infections has not been clearly identified. Several studies showed that IgM natural antibodies produced innately without previous antigen exposure, or IgM antibodies produced in response to antigen exposure, play an important and possibly unvalued role in many microbial infections, as well as in the non-inflammatory clearance of apoptotic cells (222). IgM- and complement-mediated opsonization of apoptotic cells allows fast absorption by macrophages. If this process does not occur, cells become necrotic inducing inflammation (223). Although the cellular origins of natural (innate) and immune (adaptive) IgM are different, only minimal differences in the molecular characteristics between natural and immune IgM are identified (224). Natural IgM has low affinity and high avidity (polyreactivity) and plays a paramount role in primary host defense, since it contains more flexible constitutive antigen-binding sites that enhance interactions with numerous antigens, including viruses and bacteria (224, 225). Much of the circulating natural IgM is secreted by B-1 cells, a distinct B-cell lineage that develops early during ontogeny (226). In addition, a better understanding of the production and host defense involvement of IgM has been clarified through the identification of the different roles of B-1a and B-1b cells. IgM plays a fundamental role in both, early immunity as well as long-term protection, against numerous microbial pathogens. The demonstrated generation of lasting IgM responses *in vivo* suggest that IgM production can be targeted as part of vaccination strategies (220, 224).

The pentameric structure of IgM that provides a high valency to the molecule, and the low affinity of IgM polyreactive natural antibodies, both favor agglutination (100-10,000 times more effective than IgG), which is considered as a paramount component of the IgM-mediated virus neutralization. The high valency makes pentameric IgM a more efficient immunoglobulin in the binding and removal of viral particles and other pathogens, as well as apoptotic cells (227). Most of IgM molecules are polyreactive, being fundamental components of the IgM natural antibodies, that bind with low affinity to an ample variety of different structurally non-related antigens including bacteria and viruses to which the host was never exposed (228). Polyreactive IgM monomers on B cells bind repetitive antigenic determinants on bacteria and viruses and induce natural antibody production without the involvement of T-cells. T-cell-independent type 2 antigens are identified as very repetitive structures, that include cell membrane polysaccharides and bacterial flagella, that can crosslink B cell receptors to induce an IgM response (224).

**Figure 9:**
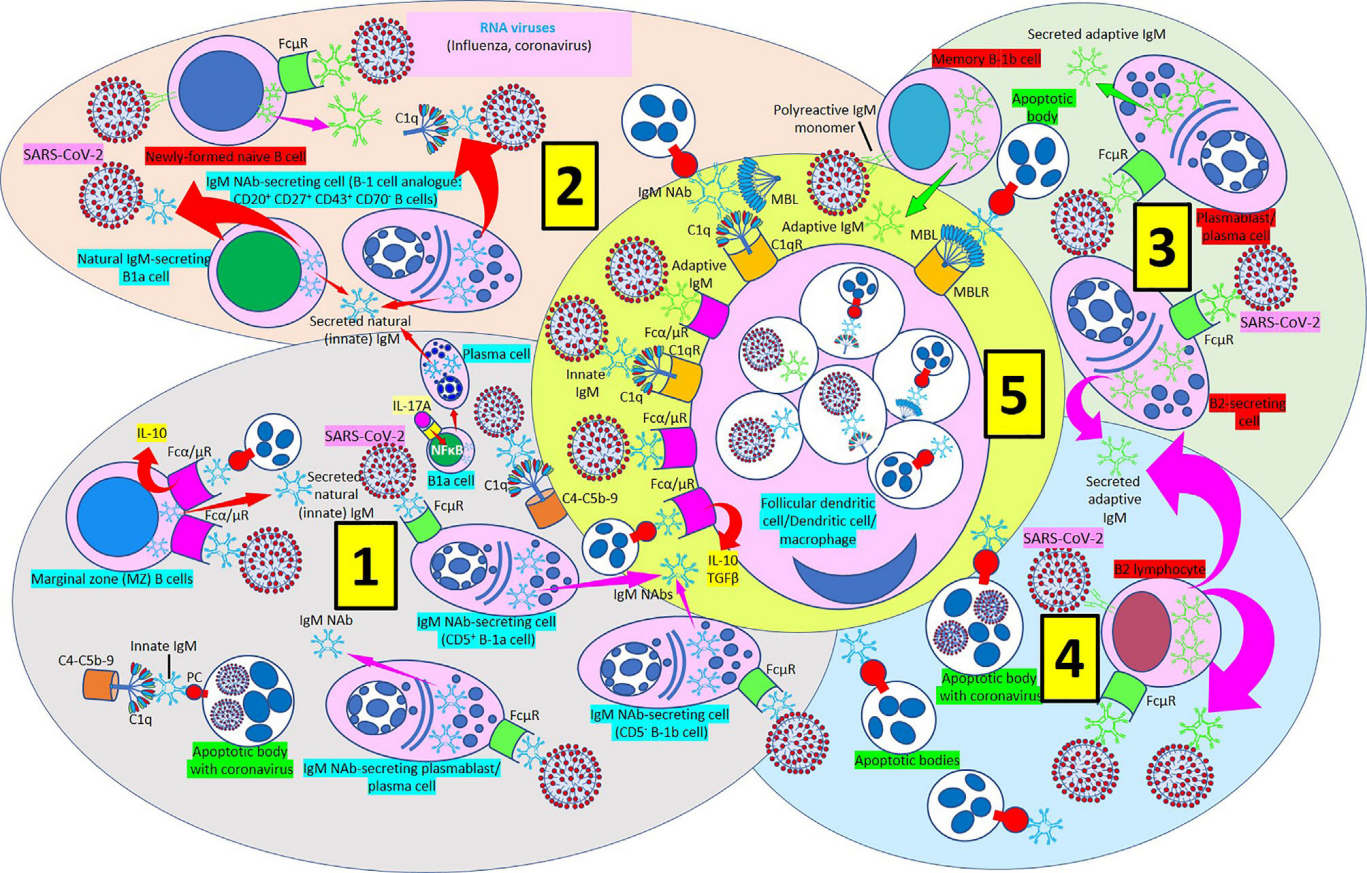
IgM-mediated immune response in coronavirus infection. [1] Most of natural (innate) immunoglobulin M (IgM) natural antibodies (NAbs) are secreted by B-1 cells (CD5^+^ B-1a and CD5^-^ B1b) without previous exposure to any antigen or pathogen. B-1a cells are critical in early protection during influenza infections through their IL-17A-driven differentiation into high-rate natural IgM producing plasma cells. Marginal zone (MZ) B cells can recognize IgM NAbs bound to RNA viruses and apoptotic cells through Fcα/μ receptors (Rs) and enhance interleukin-10 (IL-10) release. Innate IgM Nab-secreting cells (CD5^+^ B-1a and CD5^-^ B1b cells; plasmablasts/plasma cells) recognize virus-bound IgM NAbs through membrane FcμRs. Innate IgM NAbs facilitate lysis of viruses and virus-infected apoptotic cells through activation of the complement cascade (C1q-C5b-9). [2] Natural IgM-secreting B1a cells and human B1 cells, identified in the umbilical cord and in adult peripheral blood and expressing the novel CD20^+^CD27^+^CD43^+^CD70^-^ phenotype contribute to natural (innate) IgM secretion. Polyreactive IgM monomers on B cells (newly formed naïve B-cells, memory B1b cells) bind repetitive antigenic determinants on bacteria and viruses and induce immune (adaptive) antibody production without the involvement of T-cells. [3] Immune (adaptive) IgM are produced by adaptive B2 cells following exposure to an antigen or pathogen. B2-secreting cells and plasmablasts/plasma cells can produce immune IgM following antibody recognition by FcμRs. [4] B2 lymphocytes can secrete adaptive IgM antibodies following virus recognition by membrane IgM monomers and FcμR recognition of virus bound IgM. [5] Follicular dendritic cells, dendritic cells and macrophages can phagocytose viruses and apoptotic cells bound to innate or adaptive IgM through Fcα/μRs and C1qRs and enhance IL-10 and transforming growth factor (TGF) ß release to reduce inflammation.

There are two classes of IgM, natural (or innate) IgM, produced by innate-like B-1 cells without previous exposure to any antigen or pathogen; and immune (or adaptive) IgM, produced by both innate-like B-1 and adaptive B-2 cells following exposure to an antigen or pathogen (**Figure 9**) (224). B-1 cells, an innate-like B cell population responsible for natural antibody production and fast immune responses, are differentiated in B-1a and B-1b based on their expression or lack of expression of CD5, respectively (229). B1 cells in humans are present in the umbilical cord and in adult peripheral blood and express the novel CD20^+^CD27^+^CD43^+^CD70^-^ phenotype (230–232). Although natural and immune IgM are produced by different types of cells, both molecules show negligible differences in their molecular properties. Natural IgM contains more versatile antigen-binding sites able to facilitate ample interactions with numerous antigens (224). Natural IgM has been identified in humans and mice and comprises the majority of total circulating IgM (224). Natural IgM can bind to numerous microbial pathogens (224, 225). B-1a cells are critical in early protection during influenza infections through their IL-17A-driven differentiation into high-rate natural IgM producing plasma cells (233) (**Figure 9**). Interestingly, deficiency of IL-17A causes a severe reduction of B1a-derived natural antibody production in the respiratory tract what results in a profound alteration in the clearance of viral particles (233). Adaptive IgM adds to the humoral memory and long-term protection against infectious agents. Vaccination could potentially provoke long-term protective IgM responses (220). Innate and acquired humoral immune responses to viruses like the influenza virus are functionally recognizable immune responses (234).

Three Fc receptors (Rs) for IgM, that include Fcα/μR, polymeric immunoglobulin receptor (pIgR), and FcµR have been described (223). Interaction of IgM with pIgR results in secretory IgM formation in epithelial mucosal surfaces like intestine and lung. The Fcα/µR is able to bind IgM and IgA, and it is expressed on lymphocytes, follicular dendritic cells, and macrophages. The FcµR binds only IgM and it is expressed on T and B lymphocytes.

**Figure 10:**
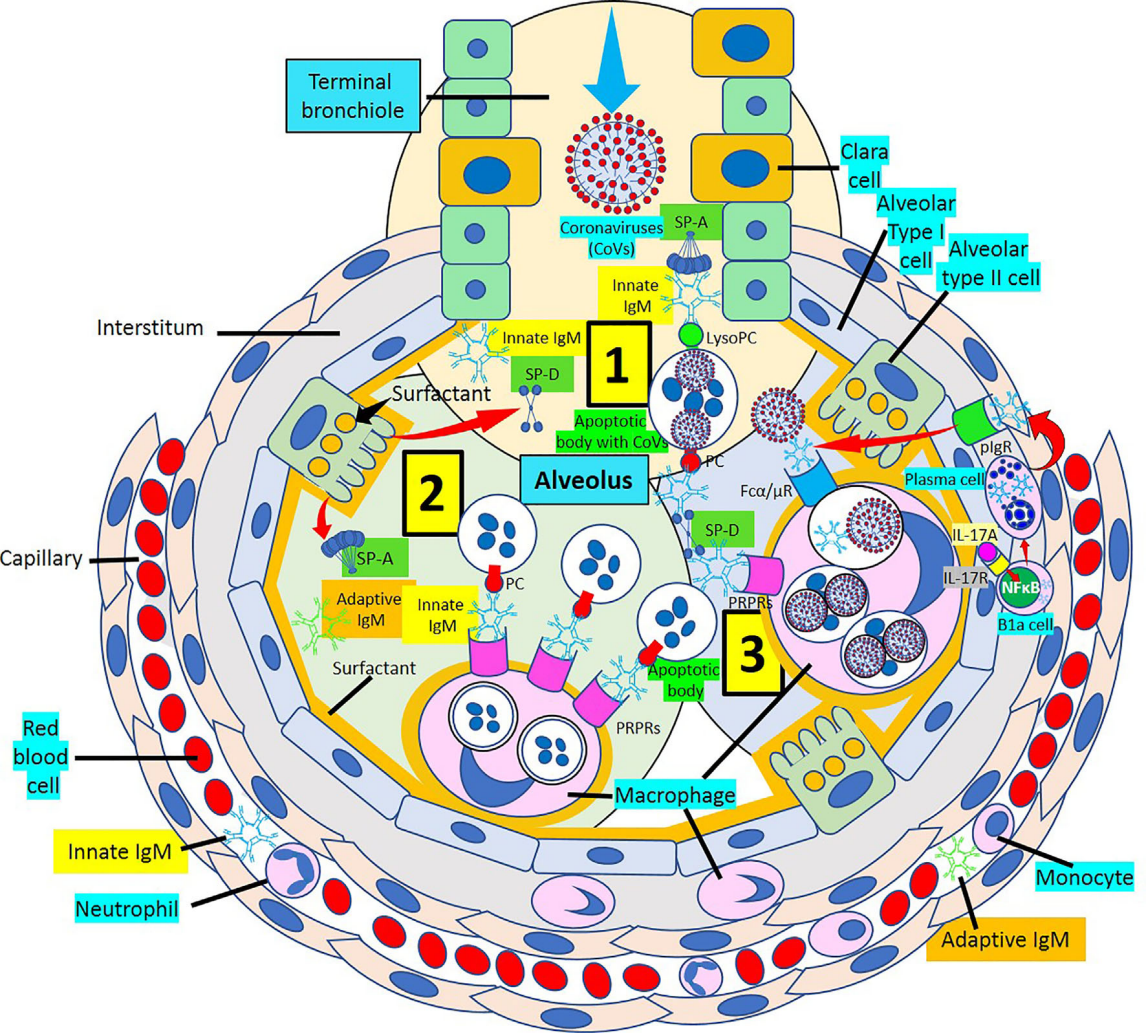
Alveolus showing IgM response to coronavirus infection. [1] The arrival of coronaviruses into the alveolus generates an immediate IgM response mediated by innate and adaptive IgM antibodies. The IgM response in association with surfactant proteins D and A (SP-D and SP-A) facilitate virus neutralization and virus-infected apoptotic cell removal. [2] Innate and adaptive IgM participate in the macrophage-mediated removal of alveolar apoptotic cells through pattern recognition protein receptors (PRPRs) avoiding inflammation. [3] Virus-infected apoptotic cells are phagocytosed with the help of SP-D and SP-A through PRPRs. Recruitment of B1a cells into the alveolar wall under the stimulation of interleukin (IL)-17A *via* IL-17 receptor (IL-17R) and nuclear factor (NF) κB activation generate plasma cells that release IgM into the wall that is transported to the alveolus *via* polymeric immunoglobulin receptors (pIgRs). Viral particles bind IgM and are phagocytosed *via* Fcα/μ receptors (Fcα/μRs). lysoPC, lysophosphatidylcholine.

IgM plays a more relevant, albeit underappreciated, role against microbial infections. IgM is very effective in the prevention and in the elimination of diverse types of microbial infections because of its unique properties (**Figure 10**). Therefore, the use of IgM to prevent and treat infections *via* immunization and/or passive antibody administration (220) has a paramount potential. It is at present very clear that a fundamental source of both natural and immune IgM comes from specialized B-1 cell subsets, but still much needs to be known regarding the origins, development, and maintenance of B-1 cell responses (225, 226, 229, 235). The knowledge obtained from research performed on B-1 responses should help in the development of vaccines designed to promote effective long-lived IgM immunity in order to prevent SARS-CoV-2 infection, improve COVID-19 disease outcome and reduce disease severity and mortality.

Each multi-cellular organism, including humans, is permanently exposed to infectious agents and transformed cells during its life cycle. Neither development nor life would be possible without an early recognition and a rapid elimination system (236). IgM NAbs behave as the ideal molecules to rapidly neutralize and remove viruses and bacteria, making them very useful molecules in the microbial innate immune responses (224). IgM has a molecular weight of 950 kDa (pentameric IgM) (237), as previously mentioned it has a high blood concentration (1.5mg/ml) (224, 237, 238), it is the first antibody triggered by an immune response after immunization or infection, and it has a serum half-life of 5 days in humans (237). IgM is generated from germline configured transcripts in B cells, has low affinity, and its high valency and ability to cause agglutination or clamping facilitates the neutralization and removal of viral particles. Due to the low affinity and high valency, the greatest number of IgM antibodies are polyreactive allowing them to bind to a wide range of phylogenetically conserved unrelated antigens present in viruses and bacteria (224).

It is generally thought that adaptive IgM does not play a significant role in long-term humoral immunity, however the identification of long-lived IgM plasma cells suggests that adaptive IgM may be an overlooked participant in humoral immunity against viral infections (220). The long-lasting contribution of IgM immune responses is supported by the demonstration that IgM immunity is not impaired and is functionally intact in the elderly (239), and perhaps due to long-lived B cells, IgM responses can be maintained for long periods of time following infection or immunization (224).

As previously mentioned, IgM antibodies are divided into natural (innate), and immune (adaptive) IgM. Natural IgM is produced by innate-like B-1 cells in the absence of previous antigen stimulation, and immune IgM is produced by both innate-like B-1 and adaptive B-2 cells after antigen stimulation. The demonstration that elevated SARS-CoV-2 specific IgM levels associate with poor outcome in patients with COVID-19 pneumonia becoming a prognostic factor for poor outcome is intriguing, and perhaps could be explained by the presence of high antibody levels indicating high viral load in these patients (240). IgM is 100–10,000 times more effective than IgG in mediating agglutination, a fundamental part of the process of IgM-mediated virus neutralization (224). The recent demonstration that intranasal administration of an engineered IgM can improve efficacy, reduce resistance and simplify the prophylactic and therapeutic treatment of COVID-19 in mice strongly suggests that enhancing the human IgM response or administering engineered IgM could significantly prevent SARS-CoV-2 infection and improve outcome (241). New research studies regarding IgM function raise the likelihood that vaccine strategies aimed at preventing virus acquisition could include this ancient weapon (220). A summary of the pattern recognition proteins proinflammatory, anti-inflammatory and antiviral effects is shown in **Table 1**.

**Table 1:** Summary of pattern recognition proteins (PRPs) proinflammatory, anti-inflammatory and antiviral effects.

Pattern recognition proteins (PRPs)	Proinflammatory effects	Anti-inflammatory effects	Effects on RNA viruses
**Surfactant protein D (SP-D)**	Trimer, monomer- CD91-CRT/OSCAR; enhance TNF-α and atherogenesis; overwhelmed inflammation	Dodecamer, fuzzy form-apoptotic cells/CD91-CRT; enhance phagocytosis; SP-D-SIRPα: anti-inflammatory	Anti-influenza through carbohydrate recognition domain-binding; virus aggregation/neutralization; hemagglutinin/neuraminidase activity (influenza) inhibition
**Surfactant protein A (SP-A)**	Abnormal clearance of apoptotic cells; Overwhelmed inflammation	SP-A-apoptotic cells/CRT-CD91; enhances phagocytosis and macrophage TGF-β	RNA virus (Influenza) agglutination and macrophage uptake; hemagglutinin inhibition
**Mannose-binding lectin (MBL)**	Overwhelmed inflammation; Abnormal macrophage uptake of apoptotic cells	MBL-apoptotic cells/CRT-CD91; enhance phagocytosis	RNA virus/apoptotic cell macrophage uptake
**Complement component 1q (C1q)**	Overwhelmed inflammation; Abnormal macrophage uptake of apoptotic cells	C1q-apoptotic cells/CRT-CD91; enhance phagocytosis	RNA virus/apoptotic cell macrophage uptake
**Native C-reactive protein (nCRP)**	Pentameric nCRP-FcγRI/FcγRIIa: increases inflammatory cytokine release; nCRP- FcγRIIb maintains a predominant anti-inflammatory effect	Pentameric nCRP bound to phosphorylcholine (PC) or lysoPC-apoptotic cells, C1q and factor H: enhance phagocytosis; nCRP-FcγRs: M2 response	Pentameric nCRP facilitates antimicrobial activity in pneumonia complicating COVID-19 infection
**Non-native CRP (nnCRP)**	Enhances inflammation and complement activation; induces atherogenesis; mostly proinflammatory	It binds atherogenic LDL, reduces foam cell formation and could also be atheroprotective	Very high CRP levels in COVID-19-associated hyperinflammation could promote nnCRP generation and inflammation
**Monomeric CRP (mCRP)**	Promotes chemotaxis; increases IL-8, MCP-1 and nitric oxide; induces ROS; mCRP-FcγRIII induce inflammation; promotes adhesion molecule expression, thrombosis and atherogenesis	Monomeric mCRP is mainly proinflammatory and not anti-inflammatory	Very high CRP levels in COVID-19-associated hyperinflammation could promote mCRP generation, inflammation, thrombosis and atherogenesis and plaque instability mediated by MMP1, 2 and 9
**Natural (innate) Immunoglobulin M (IgM)**	Lack of innate IgM response allows cell necrosis and inflammation. Lung inflammation promotes apoptotic cell clearance	Non-inflammatory clearance of apoptotic cells; enhances virus and bacteria phagocytosis	Ancient antiviral weapon. IgM-enriched intravenous immunoglobulins (pentaglobin) will enhance antiviral protection against COVID-19 and apoptotic cell removal
**Immune (adaptive) IgM**	Lack of innate IgM response allows cell necrosis and inflammation	Non-inflammatory clearance of apoptotic cells; enhances virus and bacteria phagocytosis	Ancient antiviral weapon. IgM-enriched intravenous immunoglobulins (pentaglobin) will enhance antiviral protection against COVID-19 and apoptotic cell removal

RNA, ribonucleic acid; CRT, calreticulin; OSCAR, osteoclast-associated receptor; SIRPα, signal inhibitory regulatory protein α; TGF-β, transforming growth factor-β; FcγR, Fcγ receptor; IL, interleukin; MCP-1, monocyte chemoattractant protein-1; ROS, reactive oxygen species.

## Pattern Recognition Proteins and the Bridges With Other Innate Immune Components and Adaptive Immunity

The SARS-CoV-2 pandemic is teaching us on the vital need to reestablish immune regulation to beat the virus (242). The systemic inflammation caused by massive cytokine release triggered during the viral infection involves elevated circulating cytokine levels and hyper-activation of immune system responses. This influenza-like syndrome triggers alveolar-, atherosclerotic plaque- and other organ-associated macrophages to produce an enormous amount of cytokines causing the cytokine storm and complications such as pneumonia and vascular thrombosis. As we described above, modifications of the host immune response to restore immune equilibrium is a fundamental approach to minimize disease severity and avoid death.

The immune response during a viral infection like COVID-19 involves more than the innate immunity with the participation of pattern recognition proteins. The SARS-CoV-2-associated tissue damage may be due to cytokines, acute phase physiological changes or immune-cell-mediated responses and result in severe pneumonia, intravascular coagulation and death. Marked elevations of interferon-γ, interleukin-6, interleukin-10 and interleukin-2 receptor α, a marker of T-cell activation, among others, point out the roles of inflammation and different immune response participants during COVID-19 infection (242). Although the paramount cytokine involved in the pathogenesis of COVID-19 has not been identified yet, interleukin-6 seems to be crucial (243). Inflammation limits invasive pathogens and resolves injuries by activating innate and adaptive immune responses.

**Figure 11:**
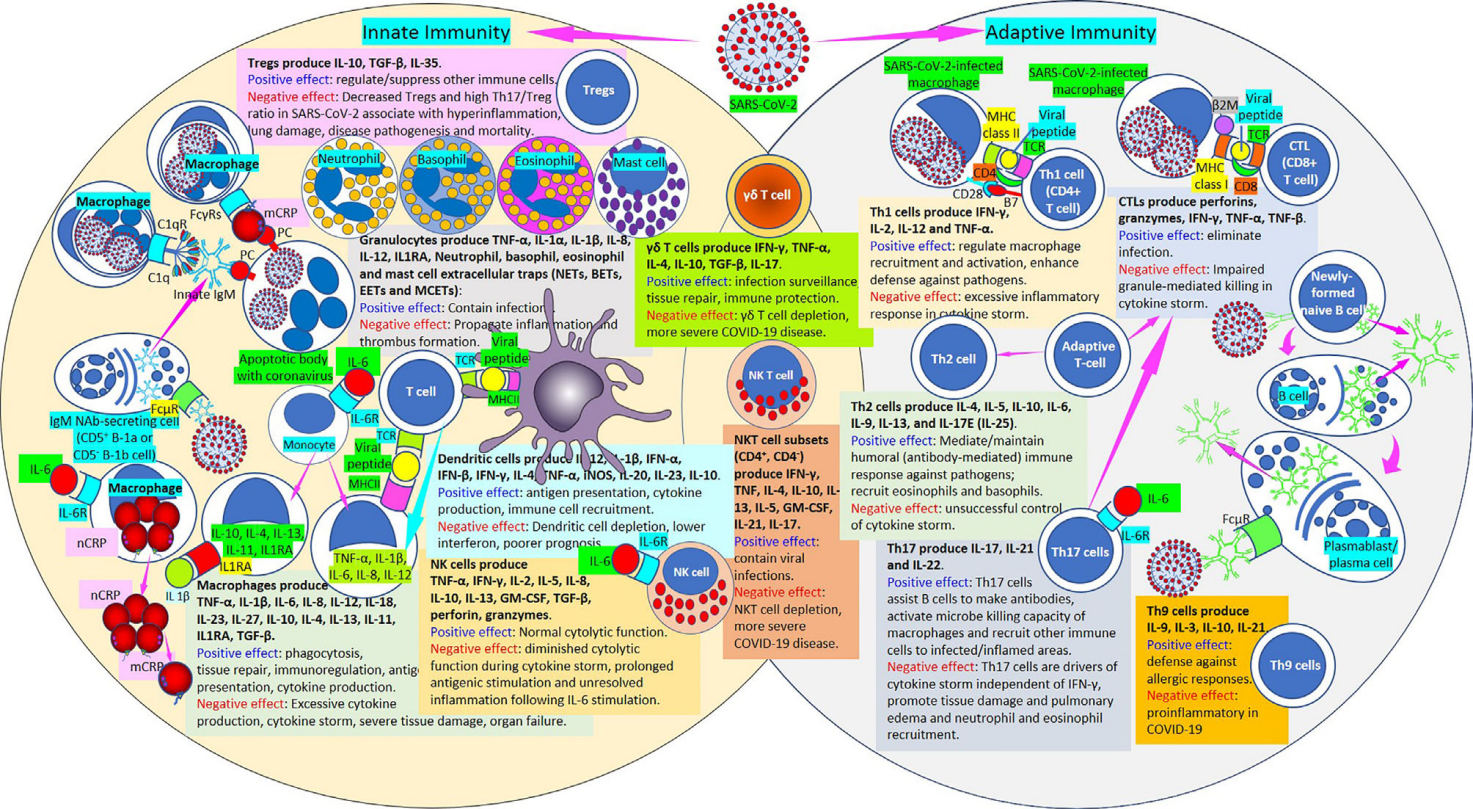
Innate and adaptive immunity in SARS-CoV-2. [Left circle] The innate immune response functions as the first line of defense against infection. It consists of soluble factors, such as native (n) and monomeric (m) C-reactive protein (CRP) produced by liver and macrophages locally; innate IgM produced by B cells; complement proteins (C1q), and diverse cellular components including granulocytes (basophils, eosinophils and neutrophils), mast cells, monocytes, macrophages, dendritic cells, regulatory T cells (Tregs) and natural killer cells. [Middle intersection] Natural killer T cells and γδ T cells are cytotoxic lymphocytes that straddle the interface of innate and adaptive immunity. [Right circle] The adaptive immune response is slower to develop, but manifests as increased antigenic specificity and memory. It consists of antibodies, B cells that produce adaptive IgM and other antibodies, Th1 CD4^+^, CTL CD8+ T lymphocytes, Th2 cells, Th17 cells, Th9 cells, among others. SARS-CoV-2, severe acute respiratory syndrome coronavirus; Tregs, regulatory T cells; IL, interleukin; Th, T helper; TNF, tumor necrosis factor; IL1RA, IL-1 receptor antagonist; NETs, neutrophil extracellular traps; BETs, basophil extracellular traps; EETs, eosinophil extracellular traps; MCETs, mast cell extracellular traps; FcγRs, Fcγ receptors; C1q, complement 1q; C1qR, C1q receptor; IgM, immunoglobulin M; PC, phosphorylcholine; FcμR, Fcμ receptor; IL-6R, IL-6 receptor; CRP, C-reactive protein; nCRP, native CRP; mCRP monomeric CRP; TGF-β, transforming growth factor-β; TCR, T cell receptor; MHCII, major histocompatibility antigen class II; IFN, interferon; NK cell, natural killer cell; NKT cell, natural killer T cell; GM-CSF, granulocyte-macrophage colony-stimulating factor; iNOS, inducible nitric oxide synthase; COVID-19, coronavirus disease-2019; β2M, β2 microglobulin; MHC class I, major histocompatibility antigen class I; CTL, cytotoxic lymphocyte.

The cells of the innate immune system are the first line of defense against microorganisms. Neutrophils, monocytes, and macrophages recognize microorganisms like SARS-CoV-2, produce cytokines, and phagocytize microorganisms and apoptotic/infected cells. Other innate immune cells, such as dendritic cells, gamma–delta T cells, and natural killer (NK) cells participate in defense mechanisms (**Figure 11**). Innate immune cells use pattern recognition receptors, which are not specific for any particular antigen, to recognize and respond to a wide variety of microbial invaders by producing cytokines that activate cells of the adaptive immune system. Macrophages normally promote phagocytosis, tissue repair, immunoregulation, antigen presentation, and cytokine production (244) (**Figure 11**). Excessive cytokine production during cytokine storm in SARS-CoV-2 infection causes severe tissue damage and organ failure (245, 246). Neutrophils produce a network of fibers containing webs of chromatin, microbicidal proteins, and oxidant enzymes called neutrophil extracellular traps (NETs) that contain infections, but when not properly regulated, NETs can propagate inflammation and vascular thrombosis (247–263). Extracellular traps are not limited to neutrophils but include eosinophils, basophils and mast cells (264) (**Figure 11**). Severe COVID-19 results in early interleukin (IL)-6, IL-10 and IL-1β-enhanced hyperinflammation (243).

This inflammatory environment is led by an abnormal function of innate immune cells that include monocytes, macrophages and natural killer cells that distribute viral pathogen-associated molecular patterns and damage-associated molecular patterns into tissues. Monocyte-derived tissue macrophages normally involved in phagocytosis, clearance of apoptotic cells, tissue repair, immunoregulation and antigen presentation, release excessive amounts of cytokines during COVID-19 infections that lead to tissue damage and organ failure (232, 233). Macrophages normally produce inflammatory molecules that eliminate microorganisms, and pattern recognition proteins like CRP, innate IgM and complement facilitate phagocytosis of infected apoptotic cells promoting tissue repair. Hyperinflammation in severe COVID-19 infection, however, causes a dysregulated macrophage response, excessive cytokine production and tissue damage (245, 246). Dendritic cells are involved in linking innate and adaptative immunity against viral infections. Dendritic cells normally participate in antigen presentation, cytokine production and immune cell recruitment; and dendritic cell dysfunction and dendritic cell depletion occur during SARS-CoV-2 infection, which are associated with lower Interferon I response and poorer prognosis. Dendritic cell changes contribute to COVID-19 pathogenesis and increased susceptibility to worst outcomes especially in the elderly (265).

Natural killer cells normally induce cytotoxicity (266, 267) and diminished cytolytic function during cytokine storm in COVID-19 disease prolong antigenic stimulation and unresolved inflammation following IL-6 stimulation (268–272). Regulatory T cells (Tregs) suppress activation, proliferation and cytokine production of CD4^+^ T cells and CD8^+^ T cells and are thought to suppress B cells and dendritic cells, being involved in immune tolerance. Tregs can produce soluble messengers which have a suppressive function, including transforming growth factor-beta and IL-10 (273, 274). The level of Tregs in severe COVID-19 disease is extremely reduced and increasing and restoring Tregs needs to be considered in order to reduce severity and perhaps improve outcomes (275–277). Gamma-delta (γδ) T cells have unique antigen recognition capacity, tissue tropism, and cytotoxic abilities that allow them to participate in infection surveillance, tissue repair and durable immune protection (278–280). Natural killer T (NKT) cells show remarkable heterogeneity among NKT cell subsets and have potent immunoregulatory functions in infectious diseases such as COVID-19 (281) since NKT cell depletion and a diminished cytotoxic potential is associated with more severe COVID-19 disease (282–284).

Normally T helper type 1 cells (Th1 cells; CD4^+^ T cells) regulate macrophage recruitment and activation enhancing defense against pathogens; Th2 cells mediate and maintain humoral (antibody-mediated) immune response against pathogens and recruit eosinophils and basophils; cytotoxic CD8^+^ T cells eliminate infection (285); Th9 cells intervene in defense against allergic responses; and Th17 cells assist B cells to make antibodies, activate the microbe killing capacity of macrophages and recruit other immune cells to infected and inflamed areas. SARS-CoV-2 infection is characterized by an excessive inflammatory response associated with a cytokine storm and a prominent lymphopenia affecting CD4^+^ T cells, CD8^+^ T cells, B cells and natural killer cells (286). Both Lymphopenia and the cytokine storm determine increased COVID-19 disease severity and enhanced mortality (286–289). Lymphopenia has been associated with high levels of IL-6, IL-10 or tumor necrosis factor (286); IL-6 seeming to play a paramount role (287, 288, 290). The severity of COVID-19 disease seems to relate to significant reduction of Th1 cells (291) and activation of Th9 cells (243) and unsuccessful control of the cytokine storm by Th2 cells. Individuals who die are more likely to have not mounted a CD4^+^ Th1 cellular response to the membrane, nucleocapsid, and spike proteins of the virus (292). Enhanced responses of Th17 cells (IL-17, IL-23) and decreased responses of Treg cells (TGF-β, IL-10) and high ratios of Th17/Treg cells in SARS-CoV-2-infected patients has a strong relationship with hyperinflammation, eosinophilic responses, allergic disorders, lung damage, and COVID-19 disease pathogenesis (293).

## Summary, Conclusions and New Treatment Horizons for Coronavirus and Other RNA Viruses

A multiweapon approach against SARS-CoV-2 infection is the best arsenal available to date to avoid development of severe COVID-19 disease and reduce mortality. The availability of vaccines that prevent severe disease, intensive care admission and death are paramount in order to reduce and prevent transmission (294). COVID-19 vaccine candidates can be grouped into three broad categories: (a) protein- based vaccines (inactivated virus vaccines, virus- like particles and protein subunit vaccines); (b) gene- based vaccines (virus- vectored vaccines, DNA vaccines and mRNA vaccines); and, (c) a combination of both protein- based and gene- based approaches (live- attenuated virus vaccines) (295). Vaccine-induced neutralizing antibodies (nAbs) are mainly produced against (1) whole virus [*Inactivated virus, PiCoVacc, Sinovac, China: nAbs* (296, 297)*; Inactivated virus, BBIBP-CorV, Sinopharm, China: nAbs* (298–300)]; (2) S1-protein receptor binding domain [*Virus vector Ad5, CanSino Biological: nAbs and T_H_1 responses* (301, 302)*; Virus vector ChAdOx1, AstraZeneca: nAbs* (303, 304)*; Virus vector Ad26 and Ad5, Gamaleya Research, Russia: nAbs and T-cell responses* (305)] and (3) S1-protein with two proline substitutions at residues K986 and V987 [*LNP-mRNA, Moderna* (306, 307) *and BioNTech with Fosun Pharma and Pfizer: nAbs and high TH1 with low TH2 responses* (308, 309)*; Protein subunit CHO, Novavax* (310)*; Virus-vectored Ad26, Janssen Pharmaceuticals* (311)]. Vaccines induce neutralizing antibody and CD4^+^ and CD8^+^ T-cell responses to the viral S protein (295).

The enhancement of components of innate human immunity needs to be seriously considered as part of the immediate armamentarium to prevent the development and progression of a serious viral infection and avoid severe inflammation of the lungs and other organs and cytokine storm that lead to higher morbidity and mortality. This is particularly important and relevant given the COVID-19 pandemic. Prior research suggests that the effective removal of apoptotic cells is fundamental for the resolution of acute injury and stimulation of tissue repair in the lung and other organs (56). Conversely, impaired clearance of apoptotic cells renders the tissues susceptible to chronic inflammation and contributes to the pathogenesis of pulmonary and other organ diseases. Under normal circumstances, alveolar macrophages, the principal macrophage cell in the lung, has a poor capacity to clear apoptotic cells, but in an inflammatory environment, alveolar macrophages develop a phagocytic capacity similar to that of other macrophages (56). These findings therefore lead to the intriguing hypothesis that the normal lung environment suppresses phagocytosis, whereas the environment present within an inflamed lung promotes apoptotic cell clearance.

Pattern recognition proteins (PRPs) like surfactant proteins A and D, mannose binding lectin, C1q, C-reactive protein, and IgM natural antibodies function as components of the innate immune system through the recognition of pathogen-associated molecular patterns (PAMPs) (e.g., RNA viruses) and damage-associated molecular patterns (DAMPs) (e.g., apoptotic cells) and subsequent facilitation of foreign particle clearance by alveolar macrophages and other macrophages in different tissues. Interestingly, PRPs perform two different roles, either pro-inflammatory or anti-inflammatory. For example, SP-A and SP-D contribute to the innate immune response by facilitating pathogen and apoptotic cell removal enhancing the proinflammatory response to viral and other infections (56). Conversely, SP-A and SP-D inhibit macrophage proinflammatory mediators. The reason for the paradox showing opposite effects of the same molecules is associated with the orientation by which the molecules bind to their respective receptors, and the type of receptor to which they are binding. For example, N-terminal collagen domain ligation with the calreticulin/CD91 receptor complex is proinflammatory, while binding of the C terminal heads to SIRPα prevents inflammation. Thus, in the naive lung, binding of SP-A and SP-D to SIRPα on the alveolar macrophages may inhibit uptake of apoptotic cells, resulting in the inefficient digestion observed for these cells. During inflammation, this inhibitory effect is replaced by a proinflammatory effect to facilitate apoptotic cell removal (56). In the resting, noninflamed lung, the lung collectins SP-A and SP-D have dual effects. On one hand, they suppress alveolar macrophage phagocytic function through their interaction with SIRPα. On the other hand, SP-A and SP-D enhance apoptotic cell removal by opsonization of apoptotic cells and facilitation of their removal through calreticulin/CD91. The net effect allows low-level phagocytosis of apoptotic cells by resting alveolar macrophages during times of health yet maintains their resting phenotype (56).

**Figure 12:**
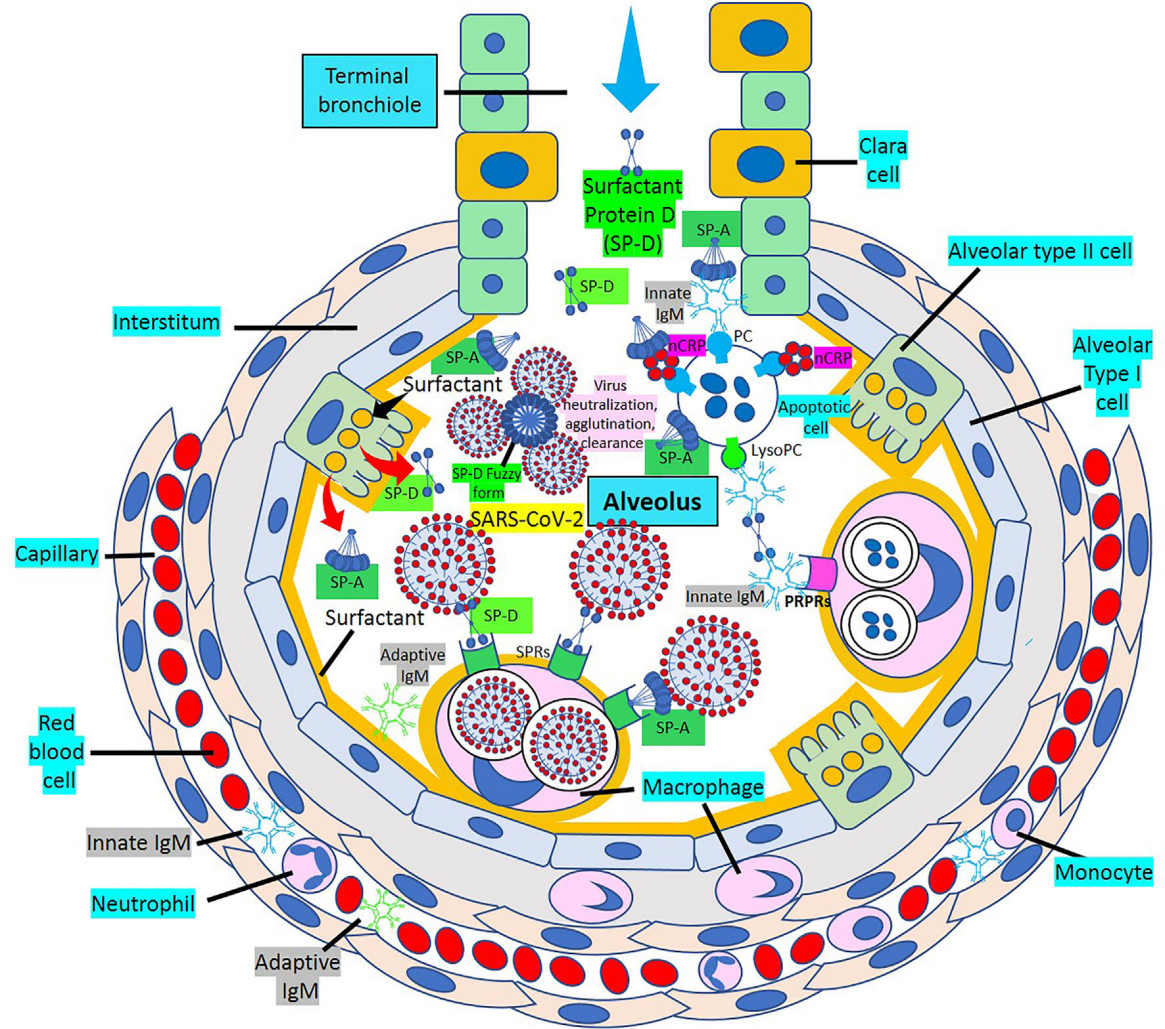
Pattern recognition protein treatment of SARS-Cov-2-related disease. Inhaled surfactant protein D (SP-D) treatment in association with endogenous SP-D and SP-A facilitates neutralization of SARS-CoV-2 particles that can be phagocytosed by alveolar macrophages *via* surfactant protein receptors (SPRs). Other pattern recognition proteins (PRPs) participate in the removal of apoptotic cells like native pentameric C-reactive protein (nCRP) bound to phosphorylcholine (PC), innate IgM/SP-A, CRP/SP-A and SP-A in apoptotic cells, in association with SP-D and innate/adaptive IgM molecules *via* pattern recognition protein receptors (PRPRs). lysoPC, lysophosphatidylcholine.

The introduction of recombinant human SP-A and SP-D suggest that pattern recognition proteins may be used as anti-infective/anti-inflammatory therapies (66, 69, 312, 313) (**Figure 12**). SP-A and SP-D (70, 71) are structurally like the viral fusion proteins they specifically bind and could be used to develop new anti-infective, and immune-modulatory therapies (70, 71). Recombinant forms of SP-D recently developed could be used therapeutically (60, 66, 67, 69, 312–314) to reduce inflammatory processes in pulmonary diseases associated with viral infections like COVID-19. Treatment with recombinant human SP-D–containing surfactant inhibits lung inflammation and enhances the resistance of surfactant to inhibition, reinforcing its potential usefulness for the prevention of lung injury in preterm newborns (314). Administration of a truncated 60-kDa fragment of human recombinant SP-D within the lung reduces the number of apoptotic and necrotic alveolar macrophages and partially corrects the lipid accumulation in SP-D-deficient mice. The same SP-D fragment binds specially to apoptotic and necrotic alveolar macrophages *in vitro*, what suggests that SP-D contributes to lung immune homeostasis by recognizing and promoting removal of necrotic and apoptotic cells (315). Therefore, SP-D administration could be another useful way to diminish apoptosis and inflammation in coronavirus-associated pulmonary disease. The observation that vascular SP-D expression may be harmful in atherogenesis implies that therapies directed to specifically inhibit low molecular weight SP-D signaling may ameliorate development of atherosclerosis, since, whereas SP-D predominantly serves as an overall host friendly innate immune molecule in both pulmonary and cardiovascular systems, low molecular weight SP-D, not multimeric SP-D appears to change into foe during development of cardiovascular disease (100, 101). Although no clinical trials treating patients with full-length recombinant SP-D have yet been performed, the body of preclinical data supports significant treatment effect (100).

The potential use of pulmonary surfactant to deliver full-length recombinant human surfactant protein SP-D (rhSP-D) using the respiratory air-liquid interface as a vehicle (316) is a promising approach to add to the arsenal against SARS-CoV-2 infection. The interaction of SP-D with surfactant constituents, the potential use of phosphatidylserine as a respiratory drug delivery system and the likelihood to produce recombinant human SP-D, brings the chance of delivering clinical SP-D-supplemented surfactants (316). Recent data highlight the therapeutic potential of a recombinant fragment of human SP-D (rfhSP-D) composed of 8 Gly-X-Y repeats, neck and CRD region against influenza and other viruses (71). This molecule binds the S1 spike protein of SARS-CoV-2 and its receptor binding domain inhibiting its interaction with the human ACE2 receptor (66, 69, 71), impeding viral entry to the host cells mediated by the ACE2 receptor following S protein cleavage by transmembrane protease serine 2 (TMPRSS2) into S1 and S2 portions (71). These data show the therapeutic potential of full-length SP-D and its fragments in SARS-CoV-2 infection (66, 69–71). As recently suggested, both recombinant SP-A and SP-D could be therapeutically relevant by blocking SARS-CoV-2 viral infection and concomitantly modulating the immune system preventing excessive inflammatory responses associated with the cytokine storm seen during COVID-19 disease (70). Interestingly, recombinant SP-A and SP-D could be therapeutically useful with current and future SARS-CoV-2 strains and the therapeutic modulation of the immunopathology caused by the COVID-19 disease (70). Previous studies showed that porcine SP-D (pSP-D) has particular influenza A antiviral activity as compared to human SP-D (hSP-D), as a result of key residues in the lectin domain of pSP-D that contribute to its profound neutralizing activity. These observations provided the basis for the design of a full-length recombinant mutant form of hSP-D, designated as “improved SP-D” (iSP-D) (66). Proteins like iSP-D could serve as novel human-based antiviral inhalation drugs against respiratory (pandemic) influenza A and SARS-CoV-2 infections in humans. Recent studies have shown that targeting the SP-A and SP-D inhibitory receptor SIRPα could be a novel approach to reduce pneumonia, a life-threatening complication in COVID-19 infection (317). Antibody blockade of SIRPα restored phagocytosis in monocytes of critically ill patients suggesting a potential strategy to prevent hospital-acquired pneumonia as a result of the SARS-CoV-2 pandemic (317, 318). The use of SIRPα antibodies have been proven effective in reducing the CD47/SIRPα inhibitory “do-not-eat-me” signal to macrophages, allowing enhanced phagocytosis of tumor cells (81). These data suggest that disrupting SIRP/CD47 macrophage signaling, either by internalization or disruption of the SIRPα structure associated with FcγR engagement, could allow macrophages to maximize their phagocytic potential against SARS-CoV-2-infected cells.

The mechanism of action most extensively reported for the antiviral effects of pattern recognition proteins relates to their ability to attach to virus particles and subsequently block virus-cell interaction. Binding of CRP to the virus and/or the cell membrane can impair subsequent virus attachment and entry into the cell (201). Besides the possible therapeutic benefits of pattern recognition proteins like CRP, changes in pattern recognition protein expression following administration of antiviral drugs and vaccines suggest the possible use of these proteins as markers of disease progression and efficacy of antiviral treatments; and CRP may be a target for the management of severe influenza diseases (201). We described above that the pentameric form of CRP, nCRP has mostly anti-inflammatory properties whereas mCRP has mostly pro-inflammatory effects (319). Interestingly, the compound 1,6-bis(phosphocholine)-hexane, a derivative of CRP-ligand phosphocholine, can prevent dissociation of nCRP, and subsequently inhibit the generation and pro-inflammatory activity of mCRP (123, 150), and can inhibit mCRP deposition and inflammation in myocardial infarction (150, 320, 321). Treatment with 1,6-bis phosphocholine-hexane reduces the inflammatory lesions in lung tissues infected with lethal influenza virus while reducing the production of CRP and reducing mortality after influenza infection (197). When we consider the properties of all CRP isoforms, it can be sustained that CRP possesses the functionality of a host defense molecule against not only atherosclerosis but against all diseases caused by proteins that behave like pathogens or toxic molecules, in a life cycle beginning as free circulatory CRP and ending in ligand-bound mCRP at sites of inflammation *via* an intermediate stage of non-native pentamers (181, 188). Although CRP inhibitor drugs are being studied for future treatment, perhaps developing better compounds that avoid the monomerization of CRP could be a more effective way to diminish tissue mCRP and inflammation (320, 321). An extracorporeal device to deplete CRP from human plasma by adsorption to beads functionalized with phosphocholine (PentraSorb CRP) has been introduced (322). It is intended for application in patients suffering from acute myocardial infarction or from other acute inflammatory disorders with elevated CRP plasma levels (322). Selective serum CRP immuno-adsorption has been shown to efficiently reduce CRP concentrations by 60% within hours (323). This approach opens a new door for possible treatment of patients with SARS-CoV-2 infection and severe disease showing very high levels of CRP.

Several strategies designed to enhance natural IgM levels have been described in recent investigations. Patients frequently develop a selective loss of circulating IgM displaying a concomitant augmented susceptibility to certain types of infections, following splenectomy or thermal injury (324). The use of Pneumococcal vaccination and i.v. administration of IgM natural antibodies (IgM NAbs) could be an alternative to neutralize viral and bacterial infections that severily increase morbidity and mortality during SARS-CoV-2 infections. Pneumococcal vaccination employs and takes advantage of the molecular mimicry that exists among the PC moieties of microbial cell-wall polysaccharide, unfractionated OxLDL, and apoptotic cells (324). Since normal human plasma contains a great amount of IgM NAbs, it may be practical and economically feasible to exploit the therapeutic potential of these IgM through production of therapeutic preparations in a way analogous to intravenous immunoglobulins that is now extensively used for the treatment of a wide range of pathological conditions. Intravenous immunoglobulins deliver effective antimicrobial activity irrespective of pathogen resistance and represent a promising alternative strategy for the treatment of diseases for which a specific therapy is not yet available. This is based on the diverse repertoire of immunoglobulins having a wide spectrum of antibacterial and antiviral specificities that are present in intravenous immunoglobulin preparations. Controlled trials, particularly with viral diseases and certain defined septic subgroups where intravenous immunoglobulins represent a promising but unproven treatment, are imperative (325). IgM-enriched Ig preparations, pentaglobin, contain 12% IgM, and were successfully used for the treatment of patients with infections associated with sepsis, as well as for transplant rejection, and for certain inflammatory experimental conditions (324).

Surfactant, a complex mixture of phospholipids and proteins that reduces surface tension at the alveolar air-liquid interface, is made up of 70-80% phospholipids, 10% surfactant proteins (SP)-A, B, C and D, and 10% neutral lipids (326, 327). It has been demonstrated that SP-D and SP-A, two pattern recognition proteins of the innate immune system (328) bind influenza A RNA viruses, inhibiting attachment and entry of the virus and also contribute to enhanced clearance of SP-opsonized virus *via* interactions with phagocytic cells (15, 329). Other pattern recognition proteins, IgM NAbs, enhance late apoptotic cell clearance in the lungs by alveolar macrophages (330). In addition, SP-D modulates the inflammatory response and helps sustain an equilibrium between effective neutralization/killing of influenza A viruses, and protection against alveolar damage resulting from influenza A virus-induced excessive inflammatory responses. Pig SP-D exhibits distinct anti-influenza A virus properties neutralizing a broad range of influenza A viruses and wild-type porcine SP-D exhibits strong antiviral properties against a much broader range of influenza A viral strains/subtypes compared to human SP-D as it is naturally expressed in the airways (15). It has been demonstrated that primary human alveolar type II cells infected with SARS-CoV, maintained under air-liquid conditions, can generate a vigorous innate immune response (331), and different cell culture systems mirroring the human airways are available, like the air-liquid interface human airway epithelium model that can be used to identify antivirals, evaluate compound toxicity and viral inhibition (332). Since IgM NAbs enhance pulmonary alveolar late apoptotic cell clearance (330), intravenous administration of IgM NAbs will intensify antiviral protection and late apoptotic cell removal in the lungs by alveolar macrophages.

The use of surfactant and SP-D as antiviral agents administered by inhalation and/or after tracheal intubation in patients requiring ventilators may offer several advantages. SP-D neutralizes a broad range of influenza A viruses and it is unlikely that a single genomic influenza A viral mutation would induce resistance against SP-D antiviral activity. Inhaled/intratracheal SP-D can provide acute protection against invading influenza A viral particles. Since SP-D is in surfactant, little toxicity and a relatively high immunogenic SP-D tolerance are anticipated in humans. Intravenous administration of IgM NAbs will enhance antiviral protection and late apoptotic cell clearance in the lungs by alveolar macrophages.

The induction of protective, long-term IgM responses may be achieved through active immunization. Vaccines involving long-term antiviral IgM responses may have advantages over conventional IgG responses against viruses that rapidly mutate, like human immunodeficiency virus and other RNA viruses that replicate by-way-of error-prone viral RNA dependent polymerases or often recombine with different viral strains (220). Considering that multimeric IgM can efficiently bind viral antigens with repeated epitopes resulting in viral agglutination, stimulating the production of these IgM antibodies could be another weapon destined to combat lethal RNA viruses like SARS-CoV-2. IgM responses have often been underestimated and assumed to be part of a transient immunity preceding the development of high-affinity IgG. IgM plays a more important role in microbial infections than has been generally appreciated, however, since IgM is highly effective in the prevention and in the elimination of numerous microbial infections. The properties of IgM allow for its utilization for prevention and treatment of infections, *via* immunization and/or passive antibody administration. A lot has been learned about both natural and immune IgM regarding their production by subsets of B-1 cells, but much still needs to be learned regarding the origins, development, and maintenance of B-1 cell responses. The knowledge acquired from the study of those responses should help with development of vaccines designed to generate effective long-lived IgM immunity (220, 223).

Finally, the success combating SARS-CoV-2 infections and COVID-19 disease needs to consider all lines of defense of the immune system. Although we emphasized the role of soluble components of the first line of defense of innate immunity like pattern recognition proteins (surfactant proteins A and D, mannose-binding lectin and complement component 1q, C-reactive protein, innate IgM antibodies), we cannot ignore the role of cellular components of the innate immune system (neutrophils, basophils, eosinophils, mast cells, monocytes, macrophages, dendritic cells, regulatory T cells, natural killer cells), cellular components of both innate and adaptive immunity (γδ T cells, natural killer T cells), soluble components of the adaptive immune system (polyreactive IgM antibodies generated in response to the viral disease, among others), and cellular components of the adaptive immune system (T cell subsets like Th1 CD4+ T cells, cytotoxic CD8+ T cells, Th2 cells, Th17 cells, Th9 cells), as depicted in **Figure 11**. **A** combined therapeutic approach that takes into account all aspects of immunity against SARS-CoV-2 virus and COVID-19 disease will allow mankind to beat this pandemic killer.

## Author Contributions

All authors listed have made a substantial, direct, and intellectual contribution to the work, and approved it for publication.

## Conflict of Interest

The authors declare that the research was conducted in the absence of any commercial or financial relationships that could be construed as a potential conflict of interest.

## Publisher’s Note

All claims expressed in this article are solely those of the authors and do not necessarily represent those of their affiliated organizations, or those of the publisher, the editors and the reviewers. Any product that may be evaluated in this article, or claim that may be made by its manufacturer, is not guaranteed or endorsed by the publisher.

## Abbreviations

RNA, Ribonucleic acid; SARS-CoV, Severe acute respiratory syndrome coronavirus; MERS-CoV, Middle East respiratory syndrome-coronavirus; H, Hemagglutinin; N, Neuraminidase; CoVs, Coronaviruses; CRP, C-reactive protein; S, Spike protein; E, Envelope protein; M, Membrane protein; N, Nucleocapsid protein; COVID-19, Coronavirus disease-2019; ACE2, Angiotensin-converting enzyme 2; ARDS, acute respiratory distress syndrome; PRP, pattern recognition protein; MBL, mannose-binding lectin; C1q, complement component 1q; nCRP, Native C-reactive protein; nnCRP, Non-native C-reactive protein; mCRP, Monomeric C-reactive protein; IgM, Immunoglobulin M; nIgM, Natural (innate) IgM; iIgM, Immune (adaptive) IgM; SP-A, Surfactant protein A; SP-D, Surfactant protein D; LRP1, Low-density lipoprotein receptor-related protein 1; CRT, Calreticulin; TGF-ß, transforming growth factor-ß; TNF-α, Tumor necrosis factor- α; OSCAR, Osteoclast-associated receptor; SIRPα, Signal inhibitory regulatory protein α; HDL, High-density lipoprotein; LDL, Low-density lipoprotein; oxLDL, Oxidized low-density lipoprotein; PC, phosphorylcholine; IgM NAbs, IgM natural antibodies.
